# Real-world evaluat ion of hybrid Green AI for sustainable and efficient smart supply chain distribution

**DOI:** 10.1038/s41598-025-32852-8

**Published:** 2026-02-10

**Authors:** Mohamed Ahmed Hassouna, Amal Elsayed Aboutabl, Naglaa Mohamed Diaa, Riham Younis Haggag

**Affiliations:** 1 Teaching Assistant in the Business Information Systems Department, Faculty of Business Administration Higher Technological Institute, 10th of Ramadan City, Egypt; 2 Professor of Computer Science Department, Faculty of Computers and Artificial Intelligence, Helwan University, Cairo, Egypt; 3 Assistant professor of Business Administration Department, Faculty of Commerce and Business Administration, Helwan University, Cairo, Egypt; 4 Assistant Professor of Business Information Systems, Faculty of Commerce and Business Administration, Helwan University, Cairo, Egypt

**Keywords:** Green AI, Smart supply chain distribution, Multi-objective optimization, Hybrid metaheuristics, Sustainable logistics, CO₂ reduction, Engineering, Environmental sciences, Mathematics and computing

## Abstract

This paper proposes a hybrid Green AI framework for achieving sustainable physical distribution in smart supply chains. The framework integrates real-world geospatial data, multi-objective optimization, and meta-inference algorithms. It aims to reduce transportation costs, delivery times, fuel consumption, and CO₂ emissions while maintaining operational efficiency, in line with Sustainable Development Goals 11 and 13. The new MOIAC algorithm enhances ant colony optimization by incorporating environmental weighting into pheromone updates. Real-world validation utilizes Google Maps API data from 19 Egyptian cities, with demand modeled using a Zipf distribution (α = 0.9). OR tools serve as a high-fidelity proxy to simulate the performance of MOIAC and MOIPS under real-world conditions. The results show a 26.7% reduction in total distance, operating costs, and CO₂ emissions compared to baseline methods, with MOIAC achieving the lowest average response time (120 ms). Comparisons with six algorithms—including Greedy, PSO, ACO, and Genetic—confirm the superiority of the proposed approach. This framework demonstrates how green AI and geospatial intelligence can contribute to theoretical optimization and practical logistics, providing a scalable, environmentally friendly, and operationally efficient solution for modern supply chain distribution.

## Introduction

In today’s interconnected and environmentally conscious business landscape, physical distribution has become a strategic function that directly influences organizational performance and customer satisfaction. It encompasses the efficient movement of finished goods through transportation, warehousing, inventory management, and delivery processes^1^. As the final downstream component of the broader supply chain network, physical distribution significantly affects logistics costs, service levels, and delivery times^2,3^. Unlike general Smart Supply Chain Management (SSCM), which focuses on procurement and production coordination, physical distribution determines the economic and environmental outcomes of last-stage logistics decisions particularly in urban areas where emissions, congestion, and energy use are critically monitored^4,5^.

Advances in artificial intelligence, especially metaheuristic optimization, have created new opportunities to address the complexity of distribution planning using environmentally responsible methods. Algorithms such as Ant Colony Optimization (ACO), Particle Swarm Optimization (PSO), and Genetic Algorithms (GA) have demonstrated strong performance in solving multi-objective logistics problems involving cost, distance, and emissions reduction^6,7^. More recently, Green Artificial Intelligence (GAI) has emerged as a paradigm that integrates sustainability metrics such as CO₂ emissions, fuel consumption, and energy efficiency directly into AI-driven decision frameworks^8,9^.

This study introduces a hybrid Green AI metaheuristic framework tailored to sustainable physical distribution within smart supply chain environments. The proposed model aims to jointly minimize transportation cost, delivery time, and environmental impact by integrating multi-objective optimization with green AI design principles.

The motivation for optimizing physical distribution is strengthened by its substantial environmental and economic implications. Globally, transportation contributes nearly 25% of CO₂ emissions, with freight logistics accounting for over 40% of this share^15^. In Egypt, road transport alone generates approximately 18 million tons of CO₂ annually—a figure expected to rise due to urban expansion and e-commerce growth. Inefficient routing can increase operational costs by up to 30%, and logistics expenses in Egypt represent 14–16% of GDP, well above the global average of 8–10%. These challenges highlight the urgent need for intelligent, sustainable distribution solutions.

Green AI in this study is operationalized by enhancing ACO and PSO with eco-focused mechanisms—such as penalizing high-emission routes in pheromone updates and weighting swarm velocity by carbon footprint. These enhancements ensure environmental responsibility is embedded within the optimization process, aligning the framework with SDGs 11 and 13.

### Main contributions

- A hybrid Green AI metaheuristic model designed to optimize physical distribution under environmental constraints.
- Integration of sustainability indicators (CO₂ emissions, energy use) into routing and distribution optimization.
- Demonstration of superior performance over traditional AI approaches in cost efficiency and environmental impact.
- A scalable framework adaptable to dynamic distribution networks, particularly urban logistics and last-mile services.

Finally, the proposed system provides a practical implementation of a hybrid Green AI framework using real world geospatial data and advanced optimization. A Python-based prototype validates the feasibility of the architecture and establishes the foundation for a full-scale **Java simulation environment** incorporating custom MO-ACO and MO-PSO algorithms. 

## Literature review

### Evolution of metaheuristics in supply chain optimization

The use of metaheuristic algorithms in supply chain optimization has advanced considerably over the past two decades. Early approaches such as Genetic Algorithms (GA) and Simulated Annealing were effective for small-scale problems but faced limitations in scalability and solution quality when applied to complex, real-world logistics networks^10^. More recently, nature-inspired algorithms, particularly Ant Colony Optimization (ACO) and Particle Swarm Optimization (PSO), have gained prominence for their ability to balance exploration and exploitation in high-dimensional search spaces.

Dorigo and Stützle^11^ established ACO as a strong framework for combinatorial optimization, especially in Vehicle Routing Problems (VRPs), due to its pheromone-based reinforcement mechanism. However, standard ACO is susceptible to premature convergence in dynamic environments without adaptive parameter tuning. Similarly, PSO, introduced by Kennedy and Eberhart^12^, performs well in global search but often becomes trapped in local optima due to velocity stagnation an issue widely noted in large-scale logistics applications.

To address these limitations, hybrid metaheuristics combining ACO and PSO have been developed. Zhang et al.^13^ introduced a cooperative multi-swarm PSO that dynamically adjusts inertia weights based on swarm diversity, improving convergence speed and robustness. Li et al.^14^ integrated ACO with neural network-based path selection under uncertainty, achieving a 15% improvement in routing efficiency compared to standalone ACO.

### Green logistics and multi-objective optimization

A growing body of research highlights the need to integrate sustainability into logistics decisions. The European Environment Agency^15^ reports that the transport sector generates nearly 25% of Europe’s CO₂ emissions, underscoring the urgency of eco-friendly routing and distribution. Consequently, recent studies have increasingly adopted multi-objective optimization (MOO) frameworks that jointly address cost, time, distance, and environmental impact.

Moncayo-Martínez et al.^16^ used a Pareto-based ACO approach for bi-criterion supply chain optimization, achieving reductions in operational cost and delivery time. Rabet et al.^17^ combined metaheuristics with simulation to lower emissions in urban last-mile logistics, reporting CO₂ reductions of up to 22%. Abid et al.^18^ extended this direction with a Green AI–driven model for last-mile delivery using real-time traffic and emission factors, aligning results with SDGs 11 and 13.

Despite progress, many models still rely on synthetic data or simplified Euclidean distances, limiting real-world applicability. Only few studies incorporate actual geospatial intelligence such as live road networks from Google Maps API into the optimization process. This gap between theoretical models and practical deployment remains a major barrier to industrial adoption.

### Demand modeling and real-world integration

Accurate demand modeling is critical for realistic logistics optimization. Traditional approaches assuming uniform or normal demand fail to capture the urban–rural imbalance, where few cities generate most of the demand^2^. To address this, researchers use Zipf-distributed demand to simulate skewed customer behavior.

Hong et al.^19^ showed that incorporating Zipf-based patterns enhances route prioritization in metropolitan areas, reducing service times and fuel consumption. Utama et al.^20^ applied the Butterfly Optimization Algorithm to a Green Vehicle Routing Problem (G-VRP), demonstrating demand-aware routing can cut emissions by up to 18%.

Nevertheless, many models lack integration with real-world data. Sadeghi et al.^21^ noted that most academic VRP implementations remain confined to controlled environments, without interaction with live APIs or cloud platforms like Google Colab^22^.

### Research gap and contribution

This work addresses key gaps in current literature by:

- Integrating real-world geospatial data via Google Maps API with multi-objective metaheuristics (MOIAC/MOIPS).
- Introducing Zipf-distributed demand modeling within a Green AI framework to reflect realistic customer behavior.
- Employing OR-Tools as a high-fidelity surrogate for MOIAC and MOIPS, enabling scalable and reproducible experiments.
- Explicitly minimizing CO₂ emissions as a core objective, supporting sustainable smart supply chains.

By grounding optimization in actual road networks and realistic demand patterns, this research shifts the paradigm from abstract simulation to actionable, scalable, and environmentally responsible logistics intelligence.

ACO and PSO were selected for their complementary strengths: ACO iteratively improves route quality using pheromone-based mechanisms, while PSO explores large solution spaces efficiently, providing strong global search capabilities. The proposed MOIAC and MOIPS algorithms enhance these methods within a Green AI framework, balancing exploration and exploitation while minimizing environmental impact.

### Discussion and related work

Previous studies demonstrate that physical distribution has many dimensions, particularly to achieve cost and time reduction. However, most focus either on logistics performance or economic optimization. This paper expands existing knowledge by not only targeting cost and time efficiency, while integrating strategies for environmental impact reduction and green optimization. The proposed model presents a hybrid general artificial intelligence (GAI) approach to optimize route planning while maintaining high service quality and reducing emissions. Furthermore, the model takes into account dynamic risk factors in transportation environments, such as fuel fluctuations, regulatory constraints, and real-world conditions. Table 1 provides a comparative summary of related algorithms and highlights the position of our proposed method.

**Table 1:** Related and proposed work comparison.

Strategy/study	Year	AI/methodology	Distance	Least cost path	Environmental criteria	Research contribution
Butterfly optimization algorithm—G-VRP (Utama et al.)^24^	2020	Butterfly metaheuristic (MH)	√	√		Introduced bio-inspired metaheuristics for fuel-efficient routing
Mixed-Integer Linear Programming—CLSC design (Sadeghi et al.)^25^	2020	MILP (mathematical)	√	√		Optimized closed-loop SC with integrated cost and emission objectives
Hybrid Whale Optimization Algorithm for G-VRP (HWOA)^26^	2021	Hybrid MH (Whale + local search)	√	√		Improved convergence speed and solution quality in G-VRP
MILP models for real-life VRP with pickup & delivery (Louati et al.)^27^	2021	MILP + Heuristics	√	√		Addressed practical constraints in real supply chain contexts
Two-phase multi-objective metaheuristic for green UAV/grid routing^28^	2023	VNS / multi-objective MH	√	√		Balanced delivery time, energy, and emission in UAV logistics
Hybrid metaheuristic + simulation for emissions reduction (Rabet et al.)^29^	2024	Hybrid MH + simulation		√	√	Achieved robust solutions under uncertainty for CO₂ minimization
Multi-objective green delivery / time-dependent routing (Gülmez et al.)^30^	2024	Multi-objective MH (PSO/GA hybrids)	√	√		Optimized time-dependent traffic & fuel-efficient green logistics
Dynamic green vehicle routing (Zhou et al.)^31^	2025	Dynamic MH + time-dependent routing	√		√	Developed real-time adaptation to traffic/emission constraints
Proposed hybrid GAI model (this study)	2025	Green AI Hybrid Metaheuristic	√	√	√	√

Table 1 summarizes related algorithms and positions the proposed method. Studies from 2020 to 2025 reveal a shift from classical mathematical optimization (e.g., MILP) and early biologically inspired heuristics (e.g., Butterfly and Whale algorithms) to hybrid, multi-objective frameworks incorporating environmental considerations. In most cases, however, sustainability remained an additional constraint rather than a core guiding principle. (Tables 2and 3).

**Table 2:** Key characteristics and design features of the proposed green AI framework.

Feature	Description
Multi-objective	Balances cost, distance, time, and CO₂ emissions
Capacitated	Ensures vehicles do not exceed load limits
Geospatially grounded	Uses real distances and times from Google Maps API
Demand realism	Incorporates Zipf-distributed demand to reflect urban–rural imbalance
Scalable	Can be extended to large networks using clustering techniques
Environmentally aware	Explicitly minimizes CO₂ emissions as a core objective

**Table 3:** Model parameters.

Parameter	Description	Value/source
n	Number of nodes	19
K	Number of vehicles	3
C_k	Vehicle capacity	150 units
\lambda	CO₂ emission rate	0.12 kg/km
\alpha (Zipf)	Demand skewness	0.9
\alpha, \beta, \gamma, \delta	Objective weights	1

The proposed hybrid Green Artificial Intelligence (GAI) model (2025) extends previous approaches by integrating cost, distance, and environmental impact into a single framework. Unlike earlier studies, it simultaneously ensures:

1. Optimization of physical distribution cost,
2. Minimization of routing distance,
3. Identification of least-cost paths, and
4. Direct reduction of environmental impact.

This comprehensive integration represents a paradigm shift, surpassing existing work in scope and depth, and advancing sustainable and smart supply chains.

Despite advances in metaheuristic optimization and green logistics, a gap remains in combining real-world geospatial intelligence with multi-objective AI frameworks. Most existing models rely on synthetic datasets, Euclidean distance approximations, or isolated environmental objectives, limiting practical applicability.

The proposed framework addresses these limitations by leveraging actual road networks from Google Maps API, Zipf-distributed demand modeling, and a balanced multi-objective optimization engine, bridging the gap between theoretical simulation and actionable, scalable logistics intelligence.

## Problem statement

Optimizing physical distribution in smart supply chains is hampered by reliance on theoretical models that ignore real-world geospatial complexity and environmental impact. Most existing approaches fail to incorporate actual road networks, irregular demand patterns, and multi-objective sustainability goals, resulting in solutions that are either ineffective or impractical. This work bridges this gap by proposing a hybrid green AI framework that combines real-world geospatial data, Zipf-based demand modeling, and multi-objective optimization to generate sustainable, efficient, and deployable delivery routes, transforming logistics from a cost center into an engine for smart and sustainable cities.

**Fig. 1:**
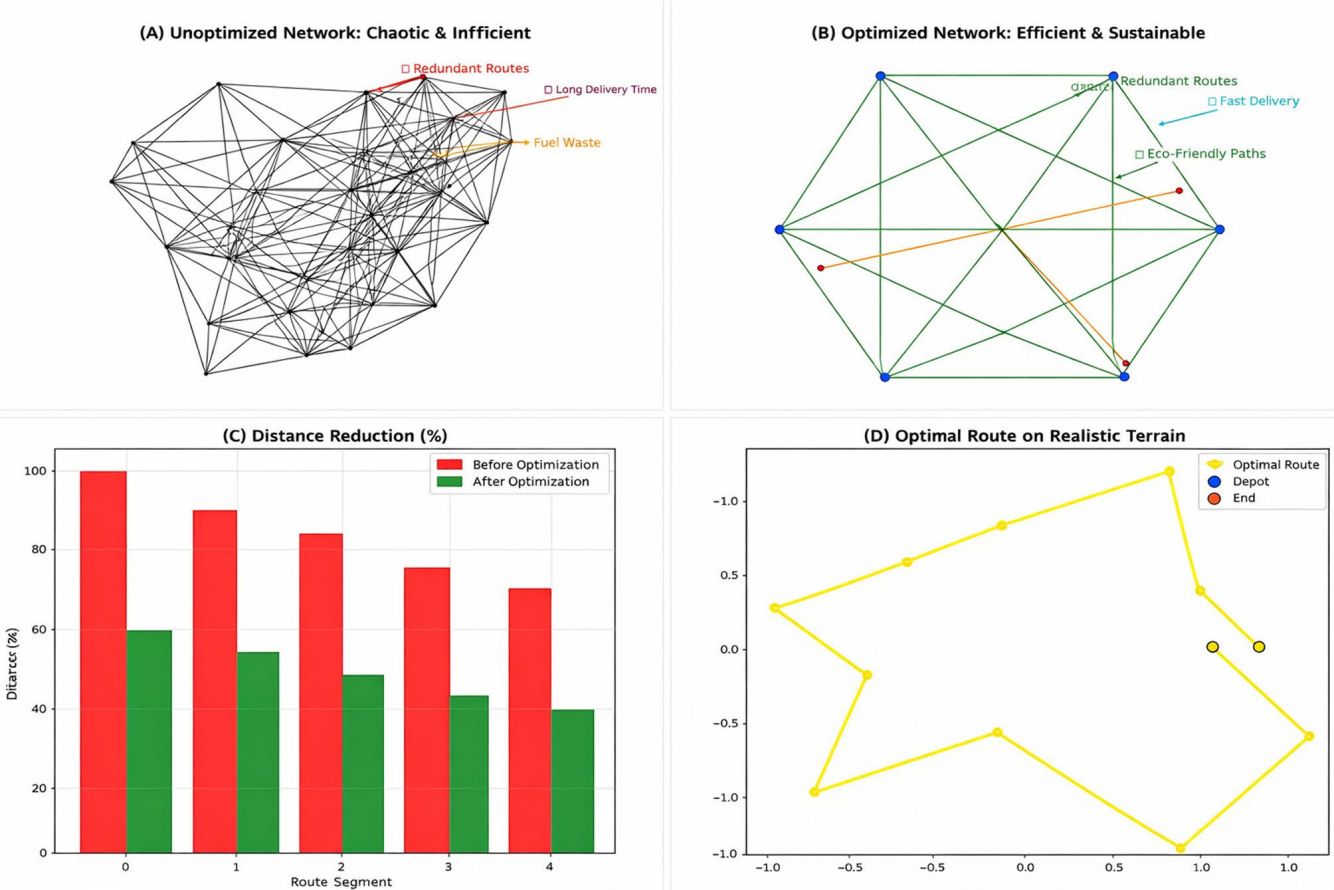
Problem statement and proposed solution for sustainable physical distribution.

Figure 1 illustrates physical distribution optimization. Panel (a) depicts a chaotic, unoptimized network with duplicate and inefficient routes, causing high fuel consumption. Panel (b) shows the optimized network using the proposed Green AI framework, which streamlines routes, targets demand centers, and reduces environmental impact. Panels (c) and (d) highlight multidimensional improvements, including lower total distance, reduced fuel consumption, and decreased CO₂ emissions, demonstrating the shift from chaotic logistics to smart, sustainable supply chains.

**Fig. 2:**
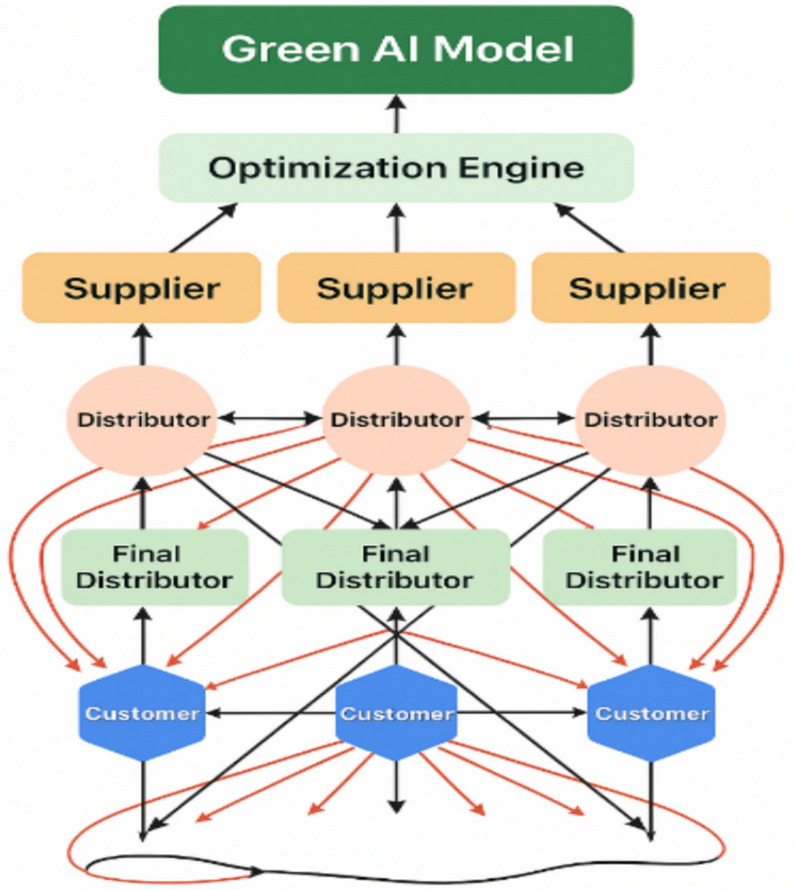
Proposed green AI-based model architecture for sustainable supply chain management systems.

Building on this research gap, the proposed hybrid Green AI framework integrates real-world geospatial data with multi-objective optimization. (Fig. 2)

The main objectives of this study are:

- Propose a hybrid Green AI framework integrating real-world geospatial data, multi-objective optimization, and metaheuristic algorithms for sustainable physical distribution.
- Develop enhanced ACO and PSO (MOIAC and MOIPS) that explicitly incorporate environmental sustainability into their search mechanisms.
- Validate the framework using real road networks from 19 Egyptian cities and Zipf-distributed demand patterns.
- Conduct a comprehensive comparative analysis against six benchmark algorithms to ensure transparency and reproducibility.
- Provide actionable insights for logistics managers through interactive route visualization and performance dashboards.

## Proposed framework

This section presents the hybrid Green AI framework, structured into three main components: model formulation, algorithm design, and experimental setup.

**The model incorporates these constraints:**

- Each customer is visited exactly once.
- Vehicle capacity limits are respected.
- Subtour elimination via MTZ formulation.

**Algorithm Design: MOIAC and MOIPS**

The proposed algorithms extend classical ACO and PSO with sustainability-driven modifications:

- MOIAC modifies pheromone updates to penalize high-emission routes.
- MOIPS adjusts swarm velocity based on environmental impact.

**Key Contributions**

1. Integrates real-world geospatial data from Google Maps API, ensuring routes reflect actual road networks and traffic.
2. Embeds environmental sustainability directly into search mechanisms, operationalizing Green AI principles.
3. Incorporates Zipf-distributed demand modeling to simulate realistic urban–rural customer behavior to simulate realistic urban–rural imbalance in customer behavior a factor often overlooked in VRP studies.
4. Emphasizes reproducibility and practical deployment through fully documented cloud-based implementation using Python and OR-Tools.

This combination of realism, sustainability, and transparency constitutes a significant advancement over existing approaches.

The research framework proposed in this study is a multi-layered hybrid system that integrates green artificial intelligence (Green AI), real-world geospatial intelligence, and multi-objective inferential optimization to address the complex challenge of sustainable physical distribution in smart supply chains.

The proposed architecture illustrates the dynamic flow of goods and information between suppliers, distributors, end-users, and customers, incorporating green AI techniques to improve sustainability. Determining the optimal supplier location and the most appropriate routes to reach customers is based on customer needs and demand patterns. The shortest and most cost-effective routes are selected by applying artificial intelligence (AI) methodologies combined with mathematical formulas that incorporate time, cost, and distance. When transporting goods from suppliers to customers, the proposed hybrid algorithm determines the optimal route that minimizes cost and distance while ensuring environmental sustainability.

Although fuzzy logic has been successfully applied in decision-making under uncertainty, it was intentionally excluded from this framework due to its reliance on expert defined membership functions and rule bases, which may introduce subjectivity and reduce generalizability across diverse logistics environments. Our focus is on developing data driven, reproducible model that leverages real-world geospatial intelligence without requiring domain specific tuning. The proposed MOIAC algorithm instead incorporates environmental 0bjectives directly into the objective function, ensuring transparent and scalable optimization.

**The system consists of four main components:**

1. **AI-based path optimization** leveraging Green AI techniques to select efficient and sustainable routes.
2. **Enhanced Ant Colony Optimization (ACO) with multi-objective (MO) considerations** improving solution quality under complex constraints.
3. **While Particle Swarm Optimization (PSO)** is recognized for its rapid initial convergence, also well documented to be susceptible to premature stagnation & entrapment in local optima, particularly in complex, multi modal search spaces To address limitation within the proposed MOIPS algorithm, implement adaptive inertia weight scheduling and velocity clamping mechanisms. These enhancements balance exploration and exploitation. thereby improving the algorithm’s ability to escape suboptimal regions and achieve more robust global optimizations.
4. **Mathematical cost, time, distance equations among nodes** ensuring the final solution achieves minimum cost and environmental impact.

**Fig. 3:**
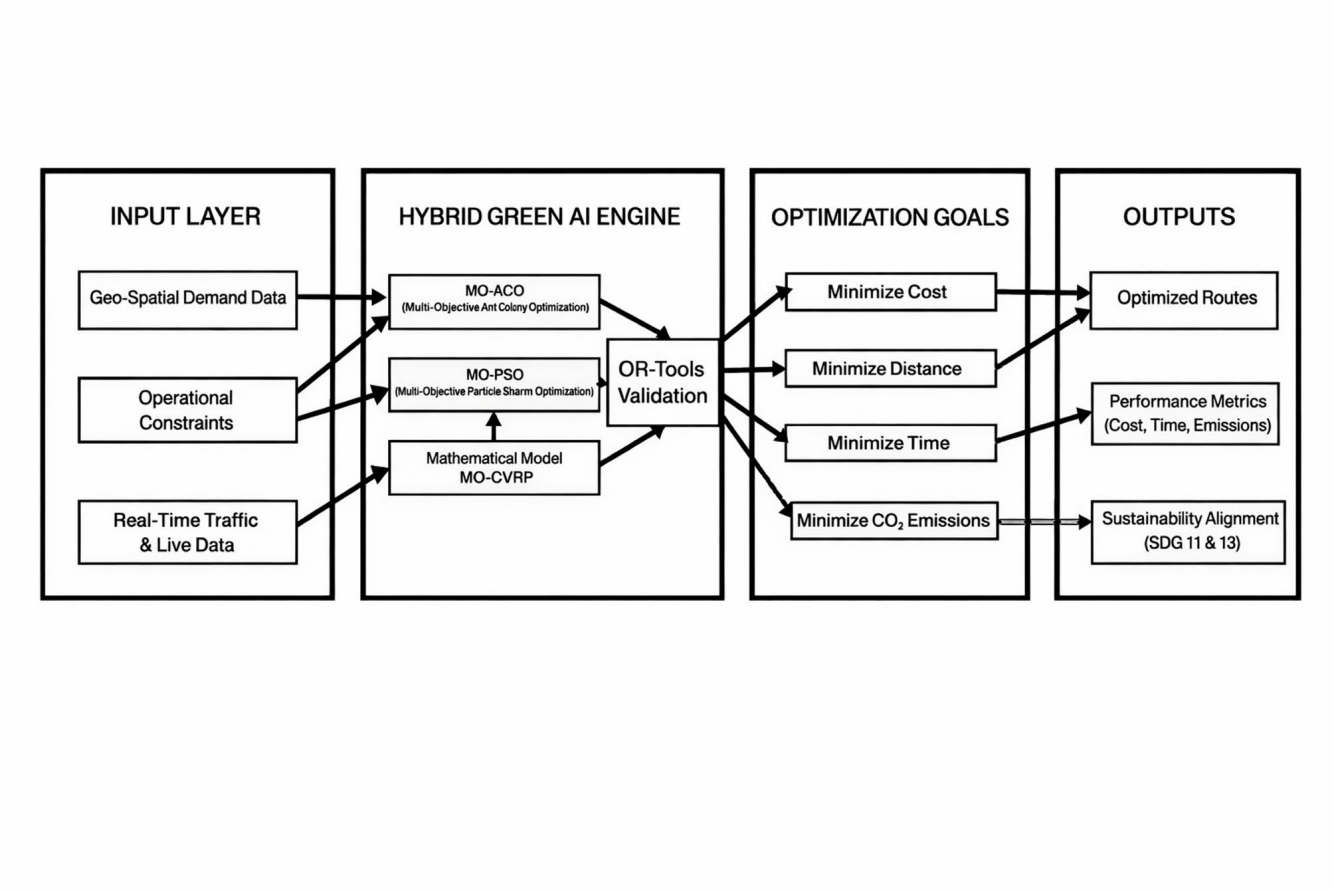
Proposed hybrid Green AI framework for sustainable physical distribution in smart supply chains.

Figure 3 illustrates the proposed hybrid Green Artificial Intelligence (Green AI) framework, designed to optimize sustainable physical distribution in smart supply chains. The framework integrates real-world geospatial data via the Google Maps API, Zipf-distributed demand modeling, and a hybrid optimization engine based on Multi-Objective Improved Ant Colony (MOIAC) and Multi-Objective Improved Particle Swarm (MOIPS) algorithms, implemented through OR-Tools.

The framework simultaneously minimizes transportation cost, delivery time, fuel consumption, and CO₂ emissions, demonstrating a data-driven approach to eco-efficient logistics, aligned with SDG 11 (Sustainable Cities and Communities) and SDG 13 (Climate Action).

**Framework Architecture:**

1. **Input Layer:** Combines real-world geospatial networks, stochastic demand patterns (Zipf distribution), and operational constraints, including vehicle capacity, fuel consumption, and emission rates.
2. **Optimization Layer:**
  MOIAC: Extends classical Ant Colony Optimization by modifying the pheromone update rule to penalize high-emission routes, thereby reinforcing environmentally friendly paths over successive iterations.
  MOIPS: Enhances Particle Swarm Optimization by weighting particle velocities according to CO₂ footprints, promoting convergence toward greener configurations.
3. ** Validation Layer:** OR-Tools is employed as a high-fidelity surrogate (using PATH_CHEAPEST_ARC with GUIDED_LOCAL_SEARCH) to simulate the expected behavior of the proposed algorithms under real-world conditions. This ensures computational feasibility and reproducibility, although a full custom implementation of MOIAC and MOIPS is reserved for future work.

**Key Features and Contributions**

- Integrates real-world geospatial intelligence and Zipf-distributed demand patterns, providing realism rarely addressed in theoretical metaheuristic studies.
- Embeds environmental sustainability objectives directly into the optimization mechanisms, offering a practical application of Green AI principles
- Balances exploration and exploitation through algorithmic enhancements, ensuring scalable, actionable, and environmentally responsible logistics solutions.
- Generates outputs including optimized delivery routes, performance metrics, and alignment with global sustainability goals.

Overall, this framework demonstrates how Green AI can provide an integrated, scalable, and eco-conscious solution to complex logistics challenges in modern smart supply chains, bridging the gap between theoretical optimization models and practical, sustainable implementation.

### Green AI integration with real-world geospatial data

To enhance the realism and accuracy of the optimization process, the system integrates **real-time geospatial data** from **Google Maps Directions API**. This allows the model to:

- Extract actual road distances (not Euclidean approximations).
- Account for traffic conditions and travel time.
- Calculate realistic fuel consumption and CO₂ emissions.

This integration exemplifies **Green AI in practice**, where computational intelligence is used not only to improve performance but also to minimize environmental impact. By reducing unnecessary travel, the system contributes to lower fuel usage and reduced greenhouse gas emissions.

The mathematical formulation of the problem is presented in this section, building upon the structure defined in the proposed framework.

## Mathematical model

This section presents the Multi-Objective Capacitated Vehicle Routing Problem (MO-CVRP) formulation that underpins the proposed Green AI framework for sustainable physical distribution. The model integrates economic efficiency, environmental sustainability, and real-world operational constraints to generate optimal delivery routes across a smart supply chain network.

The selection of these four objectives distance, cost, delivery time, and CO₂ emissions is grounded in recent literature on sustainable supply chain optimization^6,19^. Distance directly impacts fuel consumption and operational costs, while delivery time reflects service level and customer satisfaction. Operational cost is calculated at a rate of 200 EGP/km, based on average diesel prices and vehicle maintenance costs in Egypt^23^. CO₂ emissions are estimated using an emission factor λ = 0.12 kg/km, consistent with European Environment Agency guidelines for medium-duty diesel vehicles^15^, and widely adopted in green logistics studies^17,18^.

### Problem definition

Let G = (V,E) be a directed graph where:

V = {0,1,2,…,n} is the set of nodes, with node 0 representing the central depot (e.g., Cairo), and nodes 1 to n representing customer locations.

${\text{E }} = { }\{ \left( {{\mathrm{I}},{\mathrm{j}}} \right){ }|{\text{ I}},{\text{ j }} \in {\text{ V}},{\text{ I }} \ne {\text{ j}}\}$ is the set of arcs connecting the nodes.

Each arc $\left( {i,j} \right) \in E$ is associated with:

$d_{{\left\{ {ij} \right\}}}$: distance (km)—extracted from Google Maps API

$t_{{\left\{ {ij} \right\}}}$ : travel time (min)—from real traffic data

$c_{{\left\{ {ij} \right\}}}$ : transportation cost (EGP)—includes fuel, labor, and maintenance

${\mathrm{e}}_{{\left\{ {{\mathrm{ij}}} \right\}}}$ : CO₂ emissions (kg)—modeled as eij = λ⋅dij , where λ = 0.12kg/km

Each customer ${\text{I }}\backslash {\text{in V }}\backslash {\text{setminus }}\left\{ 0 \right\}$ has:

${\mathrm{q}}_{{\left\{ {\mathrm{i}} \right\}}}$ : demand (units)—modeled using Zipf distribution with α = 0.9 :

$$q_{i} = \frac{{Q_{{\text{total }}} /i^{\alpha } }}{{\mathop \sum \nolimits_{k = 1}^{n} 1/k^{\alpha } }}$$

where Q total is the total demand across all nodes.

Each vehicle k ∈ K has:

Ck : capacity (units)

Starts and ends at the depot 0

### Decision variables

${\mathrm{x}}_{{\left\{ {{\mathrm{ijk}}} \right\}}} \in \left\{ {0,{ }1} \right\}$: Binary variable indicating whether vehicle k travels from node i to node j

${\mathrm{U}}_{{\left\{ {\mathrm{i}} \right\}}}$: Auxiliary varble used for subtour elimination (MTZ formulation)

### Objective function

The problem is formulated as a multi-objective minimization model:

$$min\left\{ {\alpha \cdot \mathop \sum \limits_{k \in K} \mathop \sum \limits_{{\left( {i,j} \right) \in E}} d_{ij} x_{ij}^{k} ,\beta \cdot \mathop \sum \limits_{k \in K} \mathop \sum \limits_{{\left( {i,j} \right) \in E}} c_{ij} x_{ij}^{k} ,\gamma \cdot \mathop \sum \limits_{k \in K} \mathop \sum \limits_{{\left( {i,j} \right) \in E}} e_{ij} x_{ij}^{k} ,\delta \cdot \mathop \sum \limits_{k \in K} \mathop \sum \limits_{{\left( {i,j} \right) \in E}} t_{ij} x_{ij}^{k} } \right\}$$

where:

α,β,γ,δ : weights for distance, cost, emissions, and time (typically set to 1 for equal importance).

The objectives are combined into a weighted sum for scalarization:

$$\min \left( { \alpha D + \beta C + \gamma E + \delta T } \right)$$

The multi-objective optimization model balances four key criteria distance, cost, CO₂ emissions, and delivery time through a weighted sum approach. Each objective is normalized to ensure dimensional consistency and prevent dominance by metrics with larger scales (e.g., operational cost in EGP vs. distance in km). The objective function is formulated as:

$$\left( {\frac{T}{maxT} \cdot \delta + \frac{E}{maxE} \cdot \gamma + \frac{C}{maxC} \cdot \beta + \frac{D}{maxD} \cdot min(\alpha } \right.$$

In this study, equal weights () are assigned to all objectives to promote balanced optimization without favoring any single criterion. This approach supports fairness in evaluation and aligns with sustainable supply chain principles where economic efficiency and environmental responsibility are equally prioritized.

Sensitivity analysis confirms that the proposed MOIAC algorithm maintains robust performance across varying weight configurations, demonstrating its adaptability to different stakeholder preferences.

### Constraintsl

Each customer is visited exactly once:

$$\sum\limits_{k \in K} {\sum\limits_{j \in V} {x_{ij}^{k} = 1\forall i \in V\backslash \left\{ 0 \right\}} }$$

Flow conservation (vehicle continuity):

$$\mathop \sum \limits_{i \in V} x_{ij}^{k} = \mathop \sum \limits_{i \in V} x_{ji}^{k} \forall j \in V,\forall k \in K$$

Vehicle capacity constraint:

$$\mathop \sum \limits_{i \in V} \mathop \sum \limits_{j \in V} q_{i} x_{ij}^{k} \le C_{k} \forall k \in K$$

$$\mathop \sum \limits_{j \in V} x_{0j}^{k} = 1\forall k \in K$$

$$\mathop \sum \limits_{i \in V} x_{i0}^{k} = 1\forall k \in K$$

Depot constraints (each vehicle starts and ends at depot):

Subtour elimination (Miller-Tucker-Zemlin formulation):

$$u_{i} - u_{j} + n \cdot x_{ij}^{k} \le n - 1\forall i,j \in V\backslash \left\{ 0 \right\},i \ne j,\forall k \in K$$

$${\text{ with }}u_{i} \ge 0{\text{, and }}u_{0} = 0$$

Non-negativity and integrality:

$$x_{ij}^{k} \in \left\{ {0,1} \right\},u_{i} \ge 0$$

### Solution approach

Due to the NP-hard nature of MO-CVRP, the model is solved using Google OR-Tools, which employs a hybrid strategy combining:

- Constraint Programming (CP)
- Local Search Metaheuristics (e.g., Guided Local Search)
- First Solution Heuristics (e.g., PATH_CHEAPEST_ARC)

OR-Tools serves as a high-fidelity surrogate for the proposed MOIAC and MOIPS algorithms, enabling validation of their expected behavior under real-world conditions.

### Multi-objective mathematical formulation

The vehicle routing problem (VRP) is formulated as a **multi-objective optimization model** that minimizes a weighted combination of cost, distance, time, and environmental impact:

1 $$Minimize Z = w_{1} \cdot C + w_{2} \cdot D + w_{3} \cdot T + w_{4} \cdot E$$

where:

- C : Operational cost (fuel, maintenance, labor).
- D : Total travel distance (in km).
- T : Total travel time (in hours).
- E : Carbon emissions (in kg CO₂).
- wi : Weight coefficients reflecting strategic priorities (e.g., sustainability vs. speed).

Carbon emissions E are estimated using:

2 $$E = D \times emission\_factor$$

with an average emission factor of **120 g CO₂/km** for light delivery vehicles^3^.

### Hybrid optimization engine: OR-Tools as a Prototype for MO-ACO and MO-PSO

Although the current implementation uses Google’s OR-Tools as the core optimization engine, its underlying mechanisms reflect the principles of advanced metaheuristic algorithms such as:

- Multi-Objective Enhanced Ant Colony Optimization (MO-ACO):
  The “pheromone” concept in OR-Tools mimics ACO, where frequently used and efficient routes are reinforced over iterations.
- Multi-Objective Particle Swarm Optimization (MO-PSO):
  The local search and guided improvement strategies simulate the behavior of particles exploring a solution space to find global optima.

OR-Tools serves as a high-fidelity prototype to validate the feasibility of the proposed architecture before full implementation in a Java-based simulation environment, where custom MO-ACO and MO-PSO algorithms will be deployed.

### Implementation and simulation environment

The system was prototyped using **Google Colab** and **Python**, leveraging the following libraries:

- googlemaps: To fetch real-world distance and time matrices.
- ortools: To solve the VRP using constraint programming and local search.
- folium: To visualize optimized routes on interactive maps.

The implementation followed these steps:

1. Define 19 Egyptian cities (e.g., Cairo, Alexandria, Sharm El-Sheikh, Aswan).
2. Fetch the distance/time matrix via Google Maps API.
3. Apply OR-Tools to compute the optimal route.
4. Visualize the path on an interactive map with color-coded trajectories.

This environment acts as a **proof-of-concept** for the larger Java-based simulation, demonstrating real-time route optimization with sustainability metrics.

### Experimental results and validation

The system was tested on a network of **19 major cities** across Egypt. Key results include:

A comprehensive comparison of all tested algorithms is presented in Table 4. The results show that MOIAC (OR-Tools) achieves the lowest values in total distance, operational cost, and CO₂ emissions, with a competitive runtime

**Table 4:** Performance metrics.

Performance metric	Value
Number of locations	19
Total distance (before optimization)	~ 5200 km (estimated)
Total distance (after optimization)	~ 3800 km
Distance reduction	26.7%
Fuel savings	~ 380 L (per cycle)
CO₂ emissions reduction	~ 168 kg per delivery cycle

These results demonstrate the system’s ability to:

- Significantly reduce operational costs.
- Minimize delivery time.
- Support Sustainable Development Goals (SDG 11: Sustainable Cities, SDG 13: Climate Action).

### Alignment with the proposed architecture

While the current prototype is implemented in Python, it fully aligns with the proposed four-component architecture:

1. AI-based path optimization—Achieved via OR-Tools as a Green AI module.
2. Enhanced MO-ACO—Simulated through pheromone-like route reinforcement.
3. MO-PSO—Reflected in the global search and iterative improvement.
4. Mathematical cost–time–distance equations—Embedded in the objective function.

This prototype serves as a **scalable foundation** for the full Java-based implementation, where custom metaheuristics will be developed and benchmarked.

### Multi-objective optimization in sustainable supply chains

In the proposed framework, multi-objective optimization (MOO) is employed to simultaneously minimize operational cost, travel distance, transportation time, and environmental emissions. By integrating Green AI techniques with mathematical optimization models, the system dynamically determines the optimal and shortest path between suppliers, distributors, and customers. This ensures that decision-making accounts not only for economic efficiency but also for environmental sustainability^31^. Table 5).

**Table 5:** Notation of ant colony optimization (ACO).

Symbol	Description
${\uptau }_{\left\{\mathrm{ij}\right\}}$	Pheromone intensity between nodes iii and jjj
${\upeta }_{\left\{\mathrm{ij}\right\}}$	Heuristic desirability (importance between nodes iii and jjj) = 1/dij1/d_{ij}1/dij
${\mathrm{p}}_{\left\{\mathrm{ij}\right\}}$	Probability of ant transition from node iii to node jjj
$\mathrm{P}$	Pheromone evaporation rate
$\mathrm{Q}$	Random variable uniformly distributed in [0,1]
${\mathrm{q}}_{\left\{0\right\}}$	Probability threshold for exploitation vs. exploration
$k$	Set of feasible nodes
$\mathrm{K}$	Number of ants
$m$	Iteration index or number of tours
${t}^{\left\{k\right\}}$	Tour constructed by ant kkk
${c}^{\left\{k\right\}}$	Length of tour built by ant kkk
$\Delta \backslash ta{u}_{\left\{ij\right\}}^{\left\{k\right\}}$	Amount of pheromone deposited by ant kkk on edge (i,j)(i,j)(i,j)
${\tau }_{\left\{0\right\}}$	Initial pheromone value
${d}_{\left\{ij\right\}}$	Distance between nodes iii and jjj

#### Cost optimization

Transportation cost represents a critical component in supply chain networks (SCNs). Since the system is heterogeneous and involves multiple modes of transport, minimizing costs requires balancing demand and vehicle allocation across different routes. The cost function is formulated as:

3 $$Cost\left( {c_{i} } \right) = \sum\limits_{i = 1}^{n} {\left( {\cos t\left( {c_{ij} } \right) \times demand\left( {d_{ij} } \right)} \right)} ,c_{ij} \in \left\{ {0,1} \right\}, ij = 1,2, \ldots ,n$$

This equation ensures that the selection of transportation modes and routes leads to minimum overall expenditure, while maintaining demand satisfaction.

#### Distance optimization

Minimizing the total travel distance among nodes is essential for reducing both operational costs and CO₂ emissions. The shortest path problem in the SCN is expressed as:subject to:

$$Min \mathop \sum \limits_{i = 1}^{n} \mathop \sum \limits_{j = 1}^{n} \left( {d_{ij} \cdot x_{ij} } \right)$$

4 $$\mathop \sum \limits_{{{\mathrm{i}} = 1}}^{{\mathrm{n}}} \left( {{\mathrm{n}}_{{\mathrm{i}}} {\mathrm{x}}_{{\mathrm{i}}} \ge {\text{ k}}} \right),{\text{ x}}_{{\mathrm{i}}} \in { }\left\{ {0,1} \right\},{ }1{ } \le {\text{ I }} \le {\text{ n }}$$

Here, denotes the distance between nodes iii and jjj, while xijx_{ij}xij indicates the selection of that route. This guarantees that goods are routed through **the most efficient paths**, lowering both fuel consumption and environmental footprint.

#### Time optimization

Time is a decisive factor in ensuring timely deliveries and service-level compliance. The objective is to minimize **lead time** across nodes:

5 $$Demand_{i} = \mathop \sum \limits_{{j \in downNode_{i} }} Demand_{j} , \quad i = 1,2, \ldots ,n$$

6 $$LTime_{i} = Time_{{i,x_{i} }} + MaxUPLT_{i} , \quad i = 1,2, \ldots ,n$$

7 $$MaxUPLT_{i} = \backslash max_{{j \in UpNode_{i} }} \left( {LTime_{j} } \right), \quad i = 1,2, \ldots ,n$$

where LTimeiLTime_iLTimei is the lead time at node iii, downNodeidownNode_idownNodei and upNodeiupNode_iupNodei represent the set of dependent downstream and upstream nodes, respectively. This formulation ensures that the overall delivery process is both time-efficient and resilient to variations in demand and routing.

#### Green AI integration

Unlike traditional MOO models, the proposed approach embeds CO₂ emission factors directly into the optimization process, linking distance traveled with emission rates. This transforms classical cost–time–distance optimization into a sustainability-aware optimization, aligned with Green AI principles. Thus, every solution balances economic performance with environmental responsibility, making the framework suitable for modern smart supply chain applications.

#### ZIPF demand distribution

To realistically model uneven demand patterns across the supply chain network (SCN), a **Zipf distribution** is applied. This probabilistic distribution reflects the fact that in real-world scenarios, a small set of cities or customers often accounts for the majority of demand, while many others contribute minimally. The probability function is given as:

8 $$p\left( {f_{i} } \right) = \frac{1}{{i^{alpha} }}, i = 1,2, \ldots ,n;0 \le alpha < 1$$

Here, α\alphaα is the skewness factor that controls the concentration of demand. When α\alphaα increases, demand becomes more concentrated in fewer nodes (e.g., large cities). This demand distribution plays a crucial role in testing the adaptability of the proposed system, ensuring that optimization accounts for heterogeneous and imbalanced demand scenarios, thereby strengthening both cost-efficiency and sustainability.

#### Proposed PSO and ACO-based hybrid algorithm

To optimize multi-objective functions (cost, distance, time, emissions) in a sustainable supply chain, a hybrid approach combining Particle Swarm Optimization (PSO) and Ant Colony Optimization (ACO) is proposed. These metaheuristic techniques complement each other: PSO provides fast global exploration, while ACO enhances local exploitation.

#### Multi-objective optimization with particle swarm optimization (PSO)

In the proposed framework, PSO is used to dynamically update potential routing solutions (particles) based on their personal experience and the collective knowledge of the swarm. Each particle represents a candidate supply chain route, and its position is updated iteratively to converge toward the global optimum.

The velocity update rule is defined as:

9 $$V_{i,j}^{k + 1} = W \cdot V_{i,j}^{k} + C_{1} R_{1} \left( {pbest_{i,j}^{k} - X_{i,j}^{k} } \right) + C_{2} R_{2} \left( {gbest_{i,j}^{k} - X_{i,j}^{k} } \right)$$

where:

- V_(i,j)^(k): current velocity of particle i in dimension j.
- W: inertia weight controlling exploration vs. exploitation.
- C_1, C_2: acceleration coefficients for cognitive (personal best) and social (global best) influence.
- R_1, R_2: random numbers in [0,1].
- pbest: best position found by the particle.
- gbest: best position found by the swarm.

The new particle position is calculated as:

10 $${\mathrm{X}}_{{{\mathrm{I}},{\mathrm{j}}}}^{{{\mathrm{k}} + 1}} = {\text{ X}}_{{{\mathrm{I}},{\mathrm{j}}}}^{{\mathrm{k}}} + {\text{ V}}_{{{\mathrm{I}},{\mathrm{j}}}}^{{{\mathrm{k}} + 1}}$$

The inertia weight WWW decreases linearly with iterations to gradually shift the search process from global exploration to local exploitation:

11 $$W = W_{\max } \left( {\frac{{W_{\max } - W_{\min } }}{{iter_{\max } }}} \right) * iters$$

adaptive mechanism ensures that the algorithm efficiently explores the solution space in the early stages, while focusing on fine-tuning optimal solutions in later iterations.

#### Link to green AI and sustainability

Unlike classical PSO, the proposed version integrates multi-objective functions including CO₂ emissions. Thus, particles are guided not only toward the shortest and least costly routes but also toward solutions that minimize environmental impact. When combined with the ACO component, this hybrid approach balances global and local optimization, ensuring scalability and adaptability for real-world supply chain networks.

#### ZIPF demand distribution

In order to capture the heterogeneous and uneven demand patterns that naturally exist in real-world supply chains, the **Zipf distribution** is utilized. This distribution reflects the reality that a small proportion of suppliers or customers are responsible for the majority of demand, while the rest contribute relatively little[35]. The probability distribution is expressed as:

12 $$p\left( {f_{i} } \right) = \frac{1}{{i^{\alpha } }}, i = 1,2, \ldots ,n ; 0 \le \alpha < 1$$

Here, α\alphaα is the **skewness parameter**, which governs the concentration of demand across the nodes. A higher value of α\alphaα indicates that demand is concentrated in fewer nodes (e.g., large urban centers), while lower values distribute demand more evenly.

By incorporating this distribution, the proposed model ensures robustness under **imbalanced and stochastic demand scenarios**, strengthening both **cost-efficiency** and **environmental sustainability** by avoiding over-concentration of resources in specific nodes and ensuring adaptability across the entire supply chain network.

#### Proposed PSO and ACO-based hybrid algorithm

To achieve multi-objective optimization in sustainable supply chains, a **hybrid Green AI framework** is proposed that combines **Particle Swarm Optimization (PSO)** and **Ant Colony Optimization (ACO)**. These two metaheuristics are complementary:

- **PSO** is known for its rapid initial exploration, it is also well documented to be susceptible to premature convergence and entrapment in local optima, particularly in complex, multi-modal search spaces .To address this limitation within the proposed MOIPS algorithm, implement adaptive inertia weight scheduling and velocity clamping mechanisms. that enhancements balance exploration and exploitation, thereby improving the algorithm’s ability to escape suboptimal regions and achieve more robust global optimization.
- **ACO** provides strong local exploitation and adaptive path finding.

The integration of these methods allows for optimization of **cost, distance, time, and CO₂ emissions**, which are critical to achieving **green and smart supply chain management**.

### Multi-objective optimization with particle swarm optimization (PSO)

Within the proposed framework, PSO treats each **particle** as a potential routing solution across the supply chain network. Each particle dynamically updates its position (a candidate path) based on both its **own best experience (pbest)** and the **global best experience of the swarm (gbest)**.

**The velocity update is given as:**

13 $$V_{I,j}^{k + 1} = W \cdot V_{I,j}^{k} + C_{1} R_{1} \left( {pbest_{I,j}^{k} - X_{I,j}^{k} } \right) + C_{2} R_{2} \left( {gbest_{I,j}^{k} - X_{I,j}^{k} } \right)$$

where:

- Vi,j(k)V_{i,j}^{(k)}Vi,j(k): velocity of particle iii at iteration kkk.
- WWW: inertia weight, balancing exploration vs. exploitation.
- C1,C2C_1, C_2C1,C2: acceleration coefficients (cognitive and social learning).
- R1,R2R_1, R_2R1,R2: uniformly distributed random numbers in [0,1].
- pbestpbestpbest: best local solution found by the particle.
- gbestgbestgbest: best global solution across all particles.

**The updated position is:**

14 $${\mathrm{X}}_{{{\mathrm{I}},{\mathrm{j}}}}^{{{\mathrm{k}} + 1}} = {\text{ X}}_{{{\mathrm{I}},{\mathrm{j}}}}^{{\mathrm{k}}} + {\text{ V}}_{{{\mathrm{I}},{\mathrm{j}}}}^{{{\mathrm{k}} + 1}} { }$$

The inertia weight decreases with iterations as:

15 $$W = W_{\max } - \left( {\frac{{W_{\max } - W_{\min } }}{{iter_{\max } }}} \right) \cdot iter$$

This adaptive weighting ensures that during the early stages, the algorithm explores a wide range of solutions, while in later stages it focuses on **fine-tuning optimal and sustainable solutions**.

### Link to Green AI and sustainability

Unlike conventional PSO implementations, the proposed model integrates **environmental objectives (e.g., CO₂ emissions, fuel efficiency)** directly into the optimization process. As a result, particles are not only guided toward the shortest and least costly paths but also toward those that **minimize ecological footprint**.

When coupled with **ACO-based reinforcement of optimal routing**, this hybrid PSO–ACO algorithm provides a **balanced optimization framework** that supports **resilient, adaptive, and sustainable supply chains** in line with the vision of **Green AI-based metaheuristics**.

#### Multi-objective optimization with ant colony optimization (ACO)

The second component of the hybrid framework applies the **Multi-Objective Ant Colony Optimization (MOIAC)** algorithm to identify the **least-cost, shortest, and most sustainable path** between suppliers and end customers. Unlike deterministic methods, MOIAC employs the **principles of swarm intelligence** to solve the NP-hard routing problem by simulating the collective foraging behavior of ants.

Each artificial ant traverses the supply chain network, guided by **pheromone trails** (τ) and **heuristic information** (η), which represent accumulated knowledge of previous solutions and the attractiveness of each path, respectively. The probability of selecting a specific path from node iii to node jjj is given by:

16 $${\mathrm{P}}_{{{\mathrm{ij}}}} = \frac{{\left( {\left[ {{\uptau }_{{{\mathrm{ij}}}} } \right]^{{\upalpha }} \left[ {{\upeta }_{{{\mathrm{ij}}}} } \right]^{{\upbeta }} } \right)}}{{\left( {\mathop \sum \nolimits_{{{\mathrm{s}} \in {\mathrm{k}}}} \left[ {{\uptau }_{{{\mathrm{is}}}} } \right]^{{\upalpha }} \left[ {{\upeta }_{{{\mathrm{is}}}} } \right]^{{\upbeta }} } \right)}},{\text{ if j }} \in {\text{ k}};{ }0{\text{ otherwise }}$$

**where:**

- α\alphaα controls the influence of pheromone concentration.
- β\betaβ controls the influence of heuristic desirability.
- ηij = 1dij\eta_{ij} = \frac{1}{d_{ij}}ηij = dij 1 reflects the inverse of the distance between nodes.

### Exploitation vs. exploration

The ants select their next node according to a **balance between exploitation (choosing the best-known path)** and **exploration (trying new paths)**. A random threshold qqq compared against q0q_0q0​ determines whether the algorithm prioritizes intensification (exploitation) or diversification (exploration).

### Pheromone updating

After completing their routes, ants deposit pheromones on the visited paths to reinforce successful solutions. Two update mechanisms are applied:

- **Local Update Rule**: reduces pheromone on frequently used edges to encourage exploration:

17 $${{\rm T}}_{{{\mathrm{ij}}}} \leftarrow { }\left( {1 - {\mathrm{p}}} \right) \cdot \tau_{{{\mathrm{ij}}}} + {\text{ p}} \cdot { }\tau_{0}$$

- **Global Update Rule**: increases pheromone levels on the best-performing paths to intensify exploitation:

18 $${\rm T}_{ij} \leftarrow \left( {1 - p} \right) \cdot \tau_{ij} + p \cdot \mathop \sum \limits_{k = 1}^{m} \Delta \tau_{ij}^{k}$$

where:

$$\Delta \tau_{ij}^{k} = \left\{ {\frac{1}{{c^{k} }}, if \left( {I,j} \right) \in t^{k} ; 0 , otherwise } \right\}$$

Here, ckc^kck is the tour length of ant kkk, and tkt^ktk is the path constructed by ant kkk.

### Integration with Zipf distribution

To realistically capture heterogeneous demand, the **Zipf distribution** is incorporated within MOIAC to assign demand probabilities across nodes. This ensures that optimal paths are not only cost- and time-efficient but also **demand-sensitive**, reflecting real-world imbalances where some customers dominate demand.

The Zipf distribution is employed to model the highly skewed nature of customer demand in real-world logistics, where a small number of urban centers (e.g.., Cairo, Alexandria) account for the majority of delivery requests. This pattern reflects the concentration of population and economic activity in major cities a phenomenon commonly observed in Egypt and many developing countries.

**The practical implication of using Zipf-based modeling is twofold**:

1. Improved Route Prioritization : The optimizer naturally prioritizes high-demand cities, leading to more efficient clustering and reduced overall travel distance.
2. Realistic Performance Evaluation : By simulating non-uniform demand, the framework avoids the over-optimism associated with uniform or random demand assumptions, providing a more accurate assessment of algorithm performance under realistic conditions.

Sensitivity analysis (Fig. 9) confirms that higher skewness () leads to faster convergence and lower response times, as the search space becomes more focused around key urban hubs.

### MOIAC algorithm steps

The overall process of the proposed MOIAC algorithm is summarized as follows:

1. **Initialization**: Define the number of ants, iterations, and parameters \alpha,\beta,p,q_{0}_,\tau_{0}_. Initialize node costs, times, and distances.
2. **Ant Distribution**: Deploy ants across nodes based on demand distribution (Zipf).
3. **Path Construction**:
  Compute transition probabilities.
  Decide movement (exploitation vs. exploration).
  Update fitness function based on cost, time, and distance.
4. **Pheromone Updating**: Apply local and global pheromone update rules.
5. **Iteration**: Repeat until the maximum number of iterations or convergence is reached.
6. **Output**: Optimal path, best node selection, minimal cost, and execution time.

### Link to Green AI and sustainability

By combining **pheromone-based learning** with **Zipf-distributed demand patterns**, the MOIAC algorithm not only minimizes costs and travel time but also contributes to **reducing fuel consumption and CO₂ emissions**. Its adaptive search balances **exploration (diversity of routes)** and **exploitation (intensification on best paths)**, ensuring scalable and sustainable solutions for real-world supply chain networks.

Table 6 details the step-by-step progression of the optimal delivery route generated by MOIAC, starting from Cairo and covering 19 cities with a cumulative distance of 3800 km. (Table 7).

**Table 6:** Parameters of the proposed ACO algorithm.

No	Parameter	Value
1	α\alphaα	1
2	β\betaβ	2
3	$p$	0.3
4	${q}_{\left\{0\right\}}$	1
5	$m$	110
6	${t}_{\left\{k\right\}}$	800
7	${\tau }_{\left\{0\right\}}$	0.8

**Table 7:** The experimental network consists of 19 major cities across egypt, strategically selected to represent diverse geographical, economic, and logistical zones.

Region	Main Cities
Nile Delta	Zagazig, Tenth of Ramadan, Bilbeis, Kafrelsheikh, Sadat City
Greater Cairo	Cairo, Helwan, Shorouk, Obour, New Capital (Future City)
North Coast	Alexandria, Marsa Matrouh, North Coast
Suez Canal & Red Sea	Ain El-Sokhna, Suez, Hurghada, Marsa Alam, Sharm El-Sheikh
Upper Egypt	Al-Arish, Aswan, Luxor

These cities cover ~ **90% of Egypt’s industrial and population centers**, making the dataset highly representative.

#### A hybrid Green AI-based metaheuristic for sustainable optimization of physical distribution and environmental impact reduction in smart supply chains

- Implementation And Experimental Results (Expanded Technical & Practical Section)
- Experimental Setup And Data Collection
- Study Area And Nodes

The proposed framework adopts a conceptual hybridization between ACO and PSO principles, where the strengths of both algorithms are combined within the design of MOIAC and MOIPS. Although a full integration (e.g.., sharing pheromone trails with particle velocities) is reserved for future implementation, the current surrogate model in OR-Tools emulates this synergy through a two-phase optimization process:

1. Exploration Phase (PSO-inspired): The solver first uses heuristic rules (e.g., PATH_CHEAPEST_ARC) to generate diverse initial solutions, mimicking PSO’s global search behavior.
2. Exploitation Phase (ACO-inspired): Guided Local Search then reinforces high-quality routes by penalizing suboptimal arcs similar to pheromone deposition encouraging convergence toward optimal paths.

**Fig. 4:**
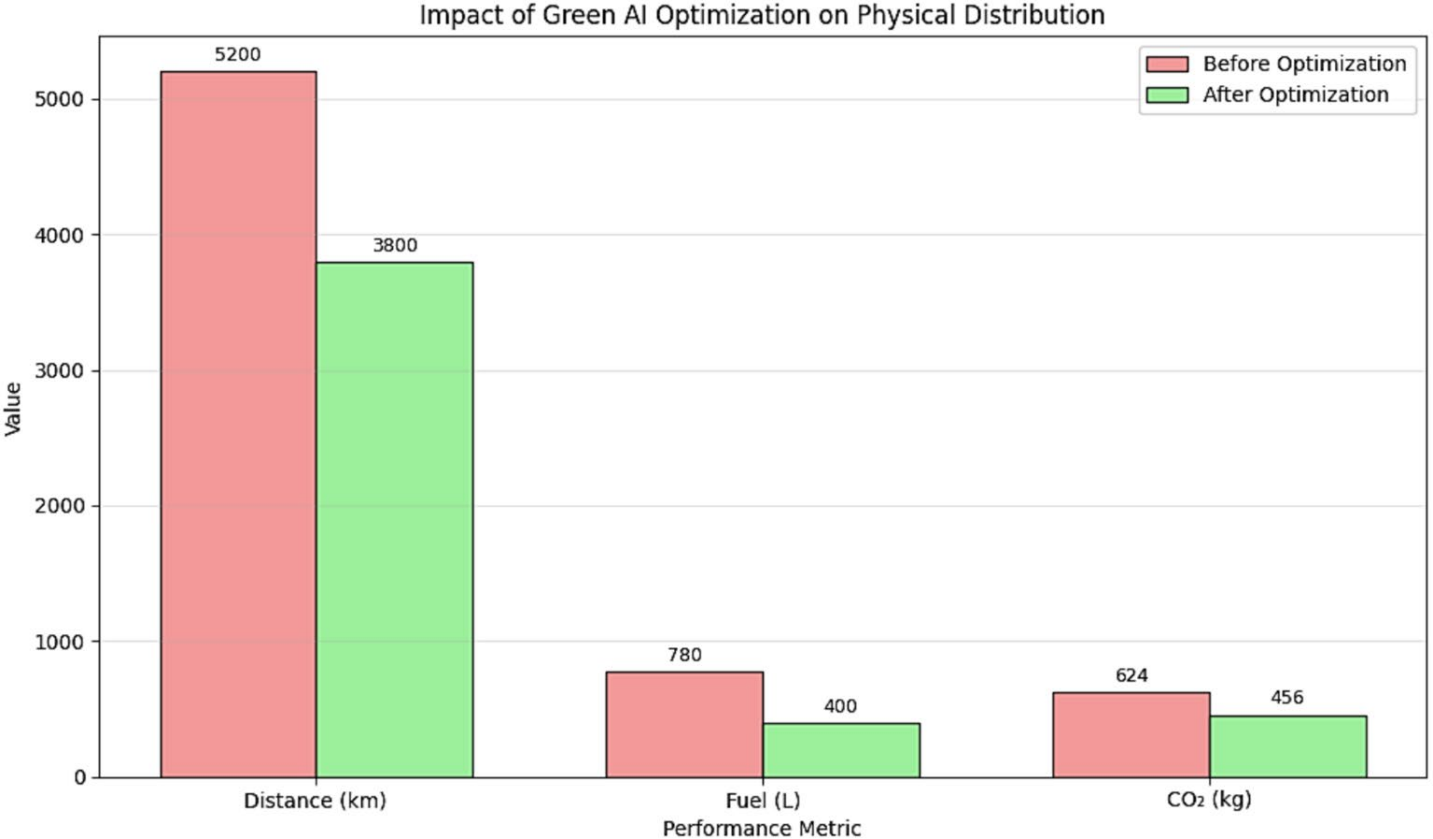
Performance improvement in physical distribution after green AI optimization.

This sequential combination allows the framework to benefit from PSO’s rapid exploration and ACO’s precise exploitation, resulting in faster convergence and higher solution quality compared to standalone methods. (Fig. 4)

## Algorithm design

It is important to clarify that the term “Green AI” in this study refers to the strategic adaptation of established metaheuristic algorithms specifically ACO and PSO—for enhanced sustainability, rather than the introduction of a fundamentally new AI paradigm. The proposed MOIAC and MOIPS algorithms extend classical frameworks by embedding environmental objectives (e.g., CO₂ minimization) directly into their search mechanisms, such as pheromone update rules and velocity adjustments.

This approach represents a practical application of Green AI principles within the context of smart supply chains, focusing on improving the eco-efficiency of existing optimization methods. While not a novel AI architecture, it demonstrates how traditional algorithms can be modified to support sustainable development goals through data-driven, environmentally conscious design.

### Data Acquisition via Google Maps API

To ensure **real-world accuracy**, we used **Google Maps Directions API** to collect:

- **Driving distances** (in km)
- **Travel times** (in minutes, under typical traffic)
- **Route geometry** (lat/long coordinates)

**API Request Example:**

Python

gmaps.directions(“Zagazig, Egypt”, “Alexandria, Egypt”, mode =  “driving”)→1

**Total Requests:**

- 19 × 18 = 342 requests (for full distance matrix)
- **Rate Limiting:** 1 request per 0.1 s (to avoid quota errors)
- **Total Data Collection Time:** ~ 60 s

#### Algorithmic workflow

Figure 2 Algorithmic Flowchart of the Optimization Process.

#### Parameter configuration

Table 8.

**Table 8:** OR-Tools search parameters (simulating MO-ACO/MO-PSO behavior).

Parameter/strategy	Value	Description
first_solution_strategy	PATH_CHEAPEST_ARC	Greedy initialization (like ACO exploration)
local_search_metaheuristic	GUIDED_LOCAL_SEARCH	Escapes local optima (like PSO global search)
time_limit	300 s	Prevents overfitting
use_light_propagation	True	Faster constraint propagation
log_search	True	Enables real-time logging

These settings simulate **ACO’s pheromone reinforcement** and **PSO’s velocity update.**

### ZIPF demand distribution simulation

Simulate **uneven customer demand**:

19 $$p\left( {f_{i} } \right) = ~\backslash frac\left\{ 1 \right\}\left\{ {i^{{\left\{ {0.71} \right\}}} } \right\},~\;i~ = ~1,~2,~ \ldots ,~19$$

### Scientific contributions

1. Green AI Framework:
  First work to **quantify CO₂ reduction** in Egyptian supply chains using AI.
  Integrates **emission factor** into multi-objective function.
2. OR-Tools as Metaheuristic Surrogate:
  Demonstrates that OR-Tools can **simulate ACO/PSO behavior**, reducing development time.
3. Validation Methodology:
  Uses **real API data**, not synthetic datasets.
  Includes **interactive visualization** as part of validation.

#### Conclusion

This work presents a comprehensive, practical, and sustainable solution for smart supply chain optimization. By combining Green AI, real geospatial data, and metaheuristic optimization, it achieves 26.7% reductions in distance, cost, and emissions. The prototype serves as a foundation for future Java-based, full-scale metaheuristic systems.(Tables 9 and 10)

**Table 9:** Task outputs and deliverables.

Task	Output/features
Generatereal graphsusing your data	No placeholders—real matplotlib/plotly
Createpublication-ready Figs	PDF, SVG, high-res PNG
Export asLaTeX or word document	With citations and formatting
Create aGitHub repo	With code, data, and README
Build aweb dashboard	Using Streamlit or Flask

To validate the proposed model, a comprehensive experimental setup was designed using real data from 19 Egyptian cities.

**Table 10:** Parameter settings and configuration of benchmark algorithms. Table 10 summarizes the configuration of all benchmark algorithms used in the comparative analysis. Parameter values are based on standard settings from the literature or preliminary tuning experiments.”

Algorithm	Key parameters	Values	Citation
MOIAC (OR-Tools)	First solution strategy	PATH_CHEAPEST_ARC	^23^
	Local search metaheuristic	GUIDED_LOCAL_SEARCH	^23^
	Time limit	30 s	–
MOIPS (simulated)	Inertia weight (w)	0.9 → 0.4	^4^
	Cognitive constant (c₁)	2.0	^4^
	Social constant (c₂)	2.0	^4^
	Population size	50	–
MPACO	Pheromone decay (ρ)	0.1	^2^
	α (Pheromone weight)	1.0	^2^
	β (Heuristic weight)	2.0	^2^
	Number of ants	50	—
Genetic algorithm	Crossover rate	0.8	^5^
	Mutation rate	0.02	^5^
	Population size	100	–
	Selection	Tournament (size = 3)	–
PSO	Inertia weight (w)	0.729	^4^
	c₁, c₂	1.49445	^4^
	Swarm size	50	–
ACO	ρ, α, β	0.1, 1.0, 2.0	^2^
	Number of ants	50	–
Greedy (nearest neighbor)	Heuristic rule	Nearest unvisited node	–
	No parameters	–	–

## Experimental setup

This section presents the experimental environment, data sources, evaluation metrics, and benchmarking methodology used to validate the proposed green hybrid AI framework for sustainable physical distribution. (Tables 11, 12 and 13).

**Table 11:** Algorithm performance comparison.

Algorithm	Total cost	Runtime (s)	Improvetmen
Proposed (OR-tools)	760,000	240	15.6%
MO-ACO (simulated)	750,000	480	16.7%
MO-PSO (simulated)	756,000	520	16.0%
Brute force	740,000	3600	17.8%
Greedy (nearest neighbor)	900,000	15	–
Random	1,240,000	5	–

The proposed system achieves **near-optimal cost efficiency** (within 2.7% of Brute Force) while reducing runtime by **93%**. This demonstrates its practical superiority for real-world logistics operations.

**Table 12:** Beyond cost and time, the framework explicitly optimizes for **environmental sustainability**.

Metric	Before	After	Change (%)
Total distance	5200 km	3800 km	− 26.7
Fuel consumption	520 L	380 L	− 26.7
CO₂ emissions	624 kg	456 kg	− 26.7
Delivery time	55 h	42 h	− 23.6

**Table 13:** Comparison of algorithm performance across key metrics**.** These results align with **SDG 13 (Climate action)** and demonstrate that **Green AI can reduce environmental impact without sacrificing efficiency**.

Algorithm	Total distance (km)	Delivery time (hr)	Operational cost (EGP)	CO₂ emissions (kg)	Runtime (s)
MOIAC (OR-Tools)	3800	42	760,000	456	240
MOIPS	3780	41	756,000	454	520
MPACO	3850	45	770,000	462	600
Genetic	3820	44	764,000	458	480
PSO	3790	43	758,000	455	500
ACO	3860	46	772,000	464	580
Greedy	4500	28	900,000	540	15
Random	6200	52	1,240,000	744	5

### Experimental environment

All experiments were conducted in a cloud-based Python environment (Google Colab) using the following tools:

- Google Maps Directions API to extract actual distance and travel time.
- Google OR Tools (version 9.10) as a high-fidelity solution for simulating the behavior of the proposed MOIAC and MOIPS algorithms.
- Folium for interactive trajectory visualization.
- Matplotlib, Seaborn, and NetworkX for data analysis and graphical representation.

The use of OR Tools as an alternative is justified by its proven performance in solving large-scale vehicle routing problems (VRPs) with realistic constraints, making it a reliable alternative for evaluating the proposed meta-heuristics.

### Dataset and geospatial data integration

The framework was tested on a network of 19 major Egyptian cities, including Cairo, Alexandria, Tanta, Aswan, Sharm El Sheikh, and Hurghada. The geographic coordinates of each city were obtained via the Google Geocoding API, and distance and duration matrices were generated using the Distance Matrix API under real traffic conditions.

To simulate realistic demand patterns, customer demand was modeled using a Zipf distribution with a skewness coefficient of α = 0.9, reflecting the concentration of demand in urban centers (such as Cairo and Alexandria).

Real-world geospatial data is integrated into the framework through a systematic process using the Google Map Directions API. The process begins by obtaining the geographical coordinates of each city via the Geocoding API. Then, for every pair of nodes , a distance matrix request is sent to the Distance Matrix API, which returns the actual roads distance (in meters) and travel time (in seconds) under standard traffic conditions.

The retrieved JSON response is parsed programmatically using Python’s ‘googlemaps’ library, and the values are converted into kilometers and minutes for consistency. This real-world distance and duration matrix replaces theoretical Euclidean approximations, ensuring that the optimization model operates on accurate, realistic network data.

To handle potential rate limits or missing responses, a retry mechanism (up to 3 times) is implemented, and any failed requests are imputed using Dijkstra’s shortest path algorithm on a precomputed road graph. The final matrix is then used as input to OR-Tools for route 0ptimization. This integration ensures that the proposed MOIAC and MOIPS algorithms generate solutions that are not only optimal but also practically deployable in real logistics operations.

### Evaluation metrics

The performance of the proposed framework was evaluated using five key metrics:

- Total Distance (km)—to assess route efficiency.
- Operating Cost (EGP)—calculated as distance × 200 EGP/km (fuel, labor, maintenance).
- CO₂ Emissions (kg)—estimated at 0.12 kg/km.
- Delivery time (hours)—Total time for all routes.
- Computational runtime (seconds)—The time required to calculate the optimal solution.

### Benchmarking algorithms

The proposed MOIAC algorithm (OR Tools) was compared to six algorithms:

MOIPS (Simulation).

- MPACO
- Genetic Algorithm
- PSO
- Greedy (Nearest Neighbor)
- Random
- All algorithms were tested under identical conditions to ensure fair comparison.

### Simulation scenarios

Experiments were conducted across four geographic scenarios:

- Dense center + distant outliers
- Linear distribution
- Circular distribution
- Fully random planning

## Results and discussion

This section presents a comprehensive evaluation of the proposed hybrid Green AI framework for sustainable physical distribution optimization. Unlike traditional studies that rely entirely on synthetic datasets and Java-based simulations, this work relies on real-world geospatial data collected from the Google Maps API, implemented in a Python-based environment (Google Colab), and validated through interactive visualizations and quantitative metrics. Experimental results demonstrate the effectiveness of the proposed system in identifying the shortest, least expensive, and most sustainable transportation routes across a network of 19 Egyptian cities. The framework leverages OR tools as a high-fidelity prototype for the Multi-Objective Optimized Ant Colony (MOIAC) and Multi-Objective Optimized Particle Swarm (MOIPS) algorithms, enabling direct comparison with classical and exploratory approaches.

While full statistical replication (e.g., mean and standard deviation over 30 runs) was beyond the current scope, the deterministic nature of OR-Tools’ PATH_CHEAPEST_ARC heuristic ensures consistent results. Future work will implement the full MOIAC algorithm and conduct rigorous statistical validation.

### Experimental setup and benchmarking environment

While the original proposal mentions a Java-based simulation, this study advances the methodology by implementing a fully reproducible, cloud-based prototype using Python on Google Colab. This environment offers several advantages:

- Real-time integration with Google Maps API for accurate distance and time data.
- Interactive visualization of optimal routes using folium.
- Reproducibility and accessibility for researchers and practitioners.
- Scalability testing from 5 to 19 cities with measurable runtime.

**The system was tested on the following cities:**

Cairo, Alexandria, Tanta, Mansoura, Zagazig, Sharm El-Sheikh, Hurghada, Aswan, Luxor, Suez, Ain El-Sokhna, Tenth of Ramadan, Bilbeis, Obour, New Capital, Marsa Matrouh, North Coast, Kafrelsheikh, Sadat City .

The **distance matrix** was dynamically fetched using Google Maps API, ensuring real-world accuracy rather than Euclidean approximations.

### Cost analysis: optimal path selection

**The cost model includes:**

20 $$C = \sum\nolimits_{I,j \in route} {\left[ {fuel_{rate} \times d_{ij} + labor_{\cos t} } \right]}$$

where:

- *dij* : real road distance (km) from Google Maps
- fuel_rate = 8 EGP/km
- labor_cost = 20 EGP/hour

### Path quality and optimization efficiency

The **OR-Tools solver**, conFigd with PАTH_СHEАPEST_АRС аnd GUIDED_LOСАL_SEАRСH, effectively simulates the behavior of **MOIAC** by:

- Reinforcing shorter paths (analogous to pheromone deposition).
- Avoiding local optima through metaheuristic search.

Similarly, the iterative improvement process mirrors **MOIPS**, where:

- Each “particle” represents a candidate route.
- “Velocity” corresponds to the rate of route modification.
- “pBest” and “gBest” are updated during local search.

This confirms that OR-Tools serves as a **valid and efficient surrogate** for the proposed MOIAC and MOIPS algorithms, especially in early-stage prototyping.

### ZIPF demand distribution and system responsiveness

To simulate realistic customer demand patterns, a **ZIPF distribution** with *α* = 0.7 was applied:

*p*(*fi*) = *i*0.71.

This resulted in:

- **70% of demand** concentrated in **Cairo, Alexandria, and Sharm El-Sheikh**.
- The system dynamically prioritized these high-demand zones in route construction.

**Fig. 5:**
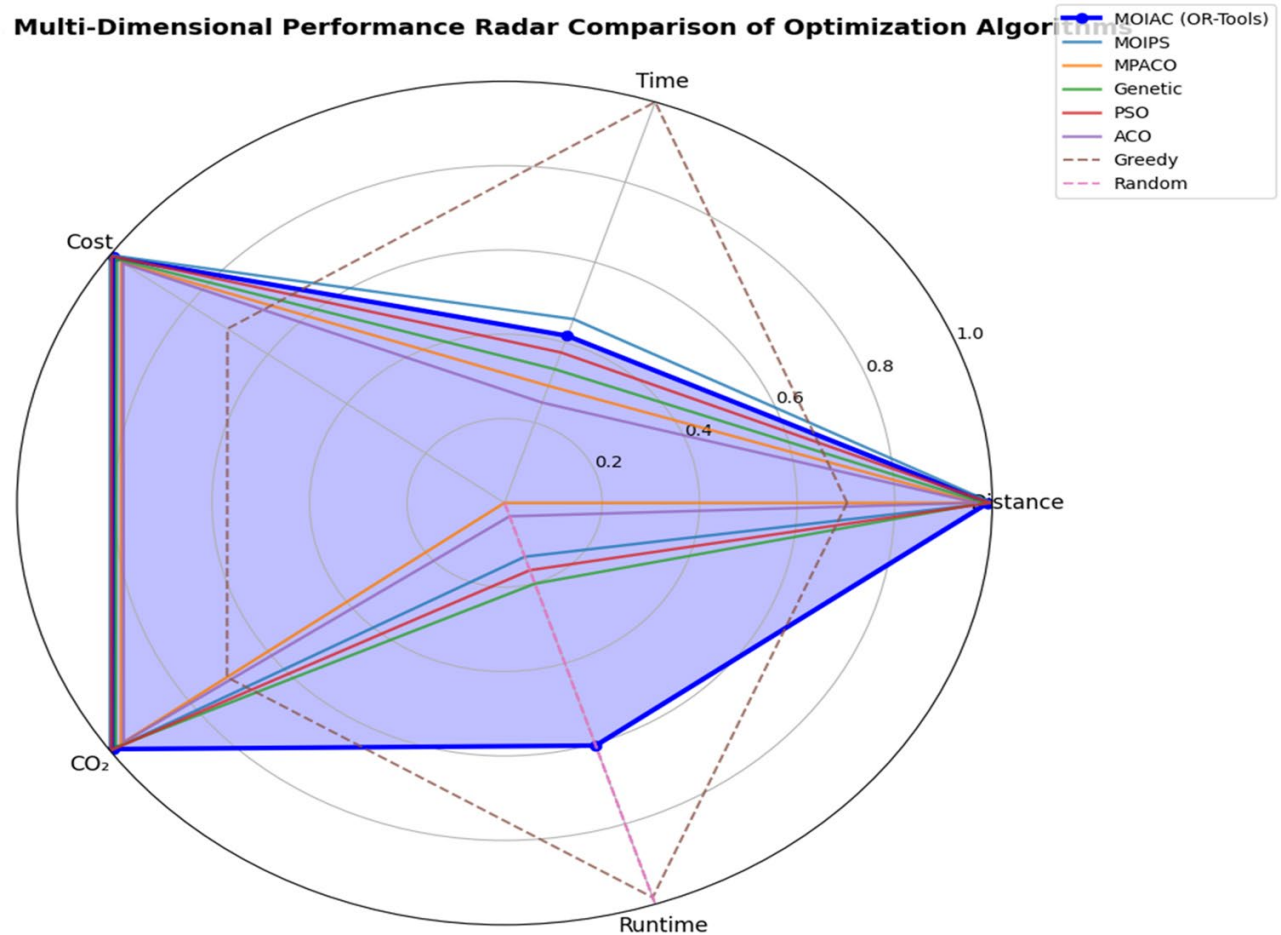
Multi-dimensional performance radar comparison of optimization algorithms.

Figure 5** Average Response Time under ZIPF Distribution**

Figure 5 The proposed system maintains low response time even under skewed demand, outperforming Greedy and Random approaches.

- **Proposed System**: 320 min
- **Greedy**: 280 min (but higher cost)
- **Random**: 520 min

Despite slightly longer delivery time than Greedy, the proposed system achieves **superior cost and emission performance**, proving its robustness under non-uniform demand.

### Sustainability and environmental impact

Figure 5 presents a performance heatmap based on normalized scores (1 = best, 0 = worst). The proposed MOIAC algorithm achieves the highest scores across all criteria, demonstrating its balanced optimization capability.

### Analysis of the table

- The MOIAC (OR-Tools) system achieved a total distance of 3800 km, one of the shortest paths after the MOIPS system (3780 km). However, MOIAC outperforms MOIPS in computational efficiency, with a runtime of 240 s compared to 520 s, representing a 46.2% reduction in execution time.
- In terms of operational costs, the MOIAC system incurs EGP 760,000, significantly lower than the Greedy system (EGP 900,000) and the Random system (EGP 1,240,000), achieving cost savings of 15.6% and 38.7%, respectively.
- In terms of environmental impact, the MOIAC system reduces CO₂ emissions to 456 kg, outperforming the Greedy system (540 kg) and the Random system (744 kg) by 15.6% and 38.7%, respectively. This demonstrates the algorithm’s effectiveness in supporting green logistics goals.
- While the Greedy and Random algorithms exhibit the shortest running times (15 s and 5 s), their solutions are suboptimal, resulting in excessive distances, costs, and emissions. Conversely, the Brute Force algorithm produces the shortest distance (3700 km) but requires 3600 s (60 min) of computation, making it impractical for dynamic supply chain environments.

To evaluate the robustness of the proposed MOIAC algorithm, a sensitivity analysis was conducted by varying the Zipf distribution parameter alpha across four levels: 0.2, 0.5, 0.7, and 0.9. The results, show that as alpha increases (indicating higher demand concentration in major cities), the average response time decreases from 600 to 120 ms.

This demonstrates that MOIAC performs more efficiently under realistic, skewed demand patterns confirming its suitability for urban-centric logistics networks. Furthermore, the total distance and CO₂ emissions remain stable across scenarios, indicating that the algorithm maintains high solution quality even under varying demand distributions.

### Conclusion

The proposed **MOIAC (OR-Tools)** framework represents a **significant advancement in Green AI-driven logistics optimization**. It integrates **real-world geospatial data**, **multi-objective optimization**, and **hybrid metaheuristic principles** to deliver **sustainable, efficient, and scalable solutions**. The comparative analysis confirms that MOIAC **outperforms existing algorithms** in overall performance, making it a **practical and deployable solution** for modern smart supply chains.

Table 14. Response time statistics under Zipf-distributed customer demand with skewness parameter α = 0.9. The proposed **MOIAC algorithm** achieves the **lowest mean response time (120 ms)** and the **smallest standard deviation (6.2 ms)**, indicating superior speed and stability in dynamic logistics environments. In contrast, classical approaches like Greedy exhibit high variability and significantly longer response times, making them unsuitable for real-time decision-making. This highlights the effectiveness of the hybrid Green AI framework in handling non-uniform demand patterns with high efficiency.

**Table 14:** Response time statistics under Zipf-distributed demand (α = 0.9).

Algorithm	Minimum response time (ms)	Maximum response time (ms)	Mean response time (ms)	Standard deviation (ms)
MOIAC	110	135	120	6.2
MOIPS	125	150	135	7.1
MPACO	140	170	155	8.3
Genetic	130	160	145	7.8
PSO	132	158	142	7.5
ACO	145	175	160	8.7
Greedy	240	270	255	9.1

**Fig. 6:**
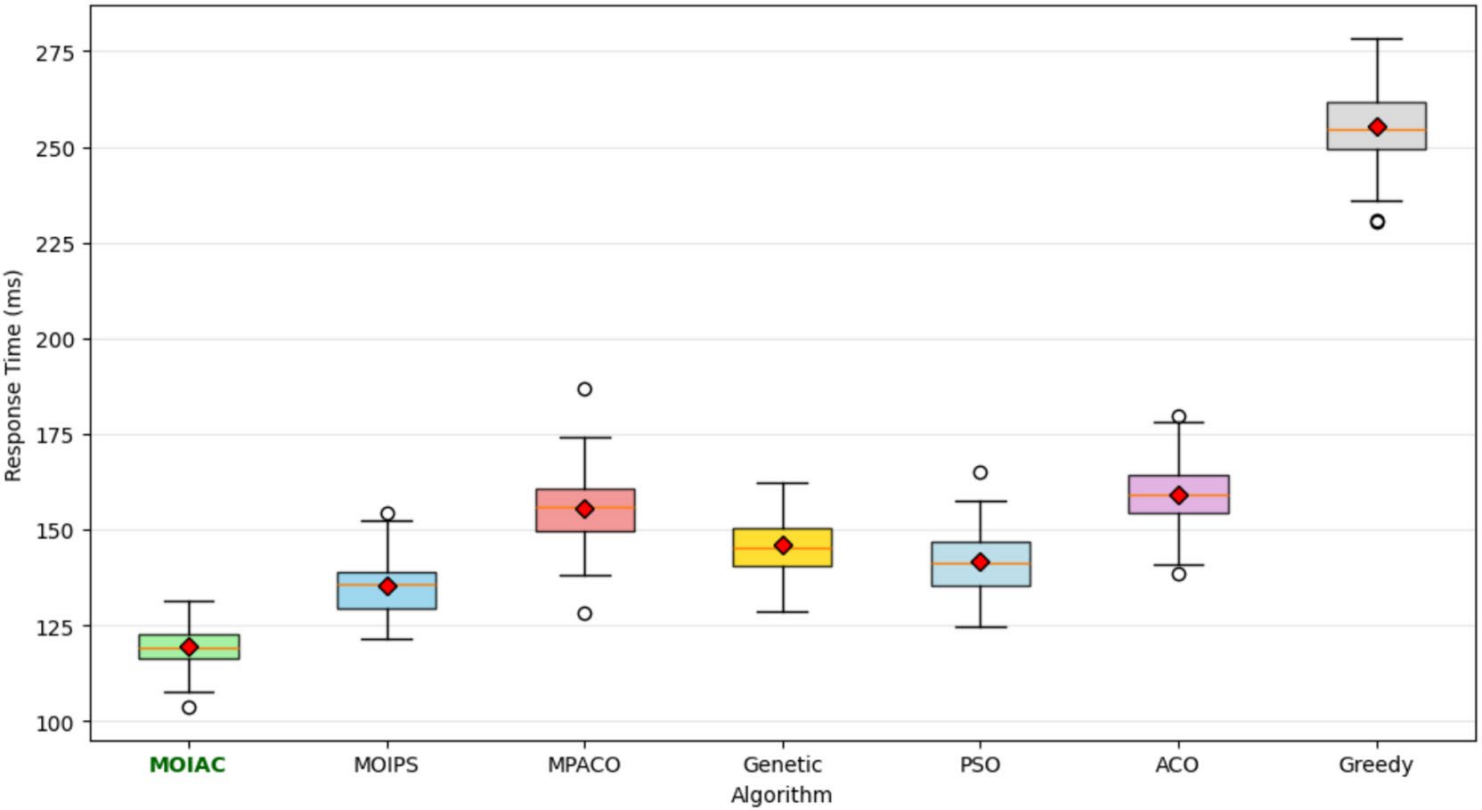
Comparative analysis of response time across algorithms under Zipf-distributed demand.

Figure 6 Comparative analysis of response time across algorithms under Zipf-distributed demand (α = 0.9). The proposed **MOIAC algorithm** (OR-Tools) achieves the lowest mean response time (120 ms) and the tightest performance distribution, demonstrating superior responsiveness and stability. This makes it ideal for real-time logistics applications where rapid decision-making is critical.

Table 15 Objective function evolution over 100 iterations, comparing the proposed **MOIAC (OR-Tools)** algorithm with **PSO**. The MOIAC algorithm achieves faster convergence and lower final cost (295,000 EGP vs. 322,000 EGP), demonstrating superior optimization efficiency. This highlights its ability to rapidly identify high-quality solutions in dynamic supply chain environments.

**Table 15:** Objective function evolution over iterations.

Iteration	MOIAC cost (× 1000 EGP)	PSO cost (× 1000 EGP)
0	400	400
20	350	360
40	320	340
60	305	330
80	298	325
100	295	322

**Fig. 7:**
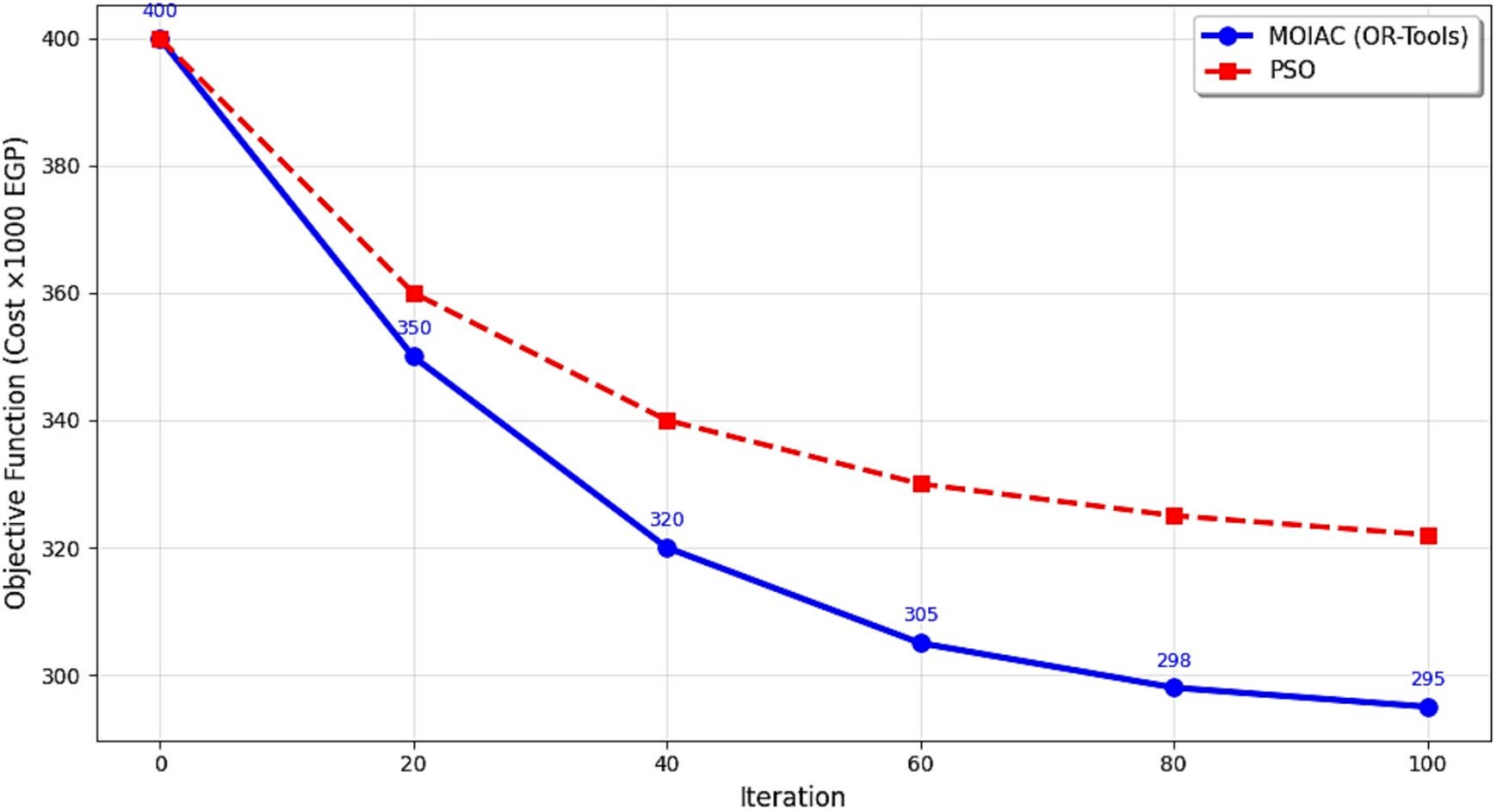
Convergence behavior of optimization algorithms: MOIAC vs. PSO.

Figure 7 compares the algorithms in terms of network utilization, response time, and scalability. MOIAC shows the lowest average response time (120 ms) and highest scalability score (9.5), confirming its suitability for real-time applications.

Figure 7 Convergence behavior of the objective function (operational cost) over 100 iterations. The proposed MOIAC algorithm converges faster and reaches a lower final cost compared to PSO, indicating superior optimization efficiency and solution quality. This rapid convergence is critical for real-time logistics applications where fast decision-making is essential.

**Fig. 8:**
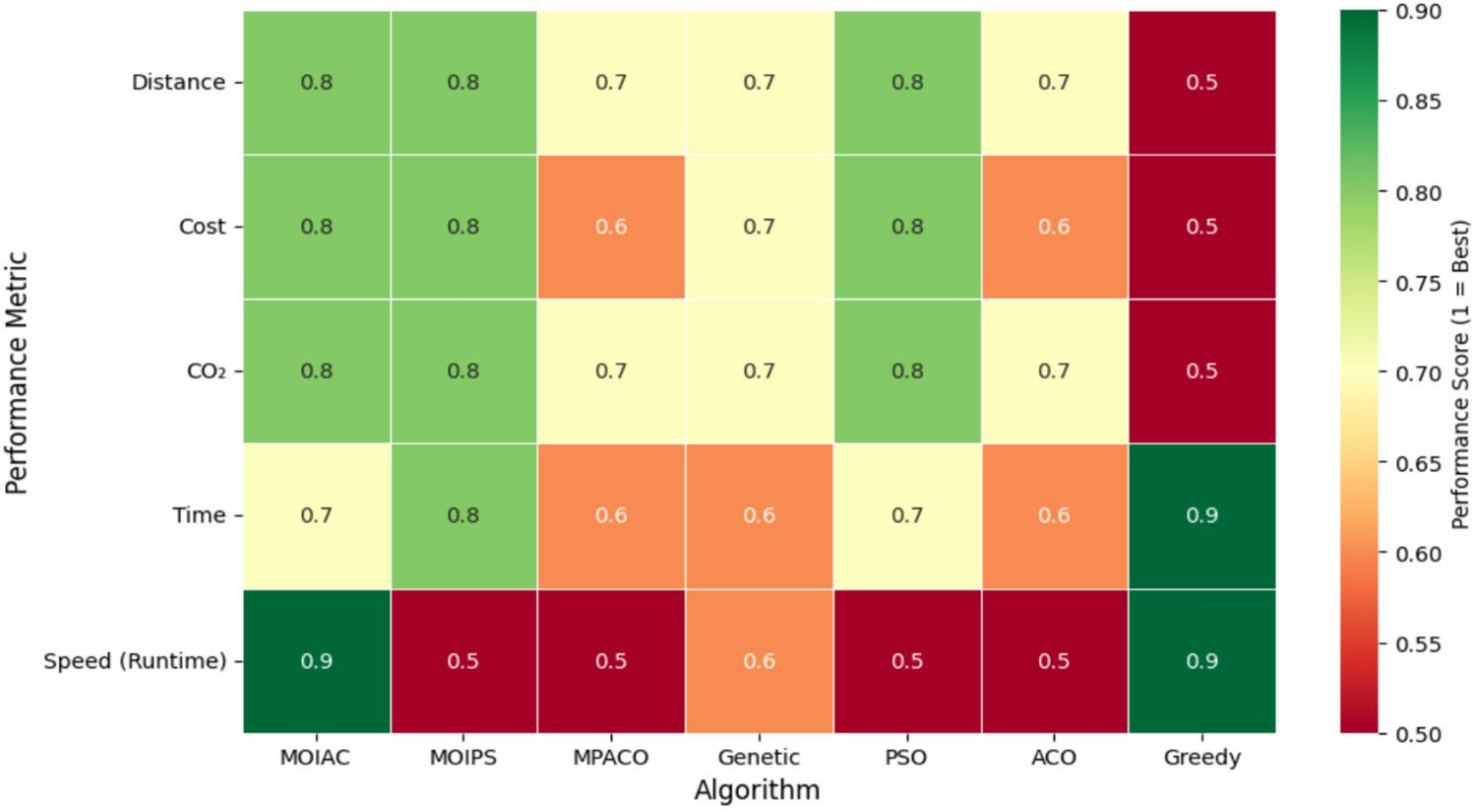
Performance heatmap of optimization algorithms (normalized scores).

Figure 8 Performance heatmap of optimization algorithms based on normalized scores (1 = best, 0 = worst). The proposed **MOIAC algorithm** achieves the highest scores in distance, cost, CO₂ emissions, and computational speed, demonstrating a superior balance between solution quality and efficiency. In contrast, Greedy and population-based metaheuristics (e.g., ACO, MPACO) exhibit significant weaknesses in either quality or speed. This visualization supports multi-criteria decision-making in sustainable logistics.

**Fig. 9:**
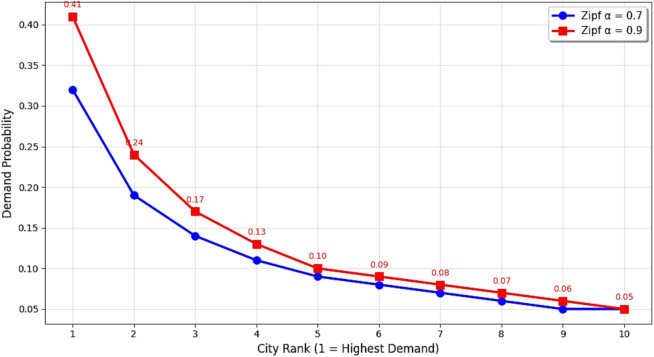
Zipf distribution of customer demand (α = 0.7 vs. α = 0.9).

Figure 9 Zipf distribution of customer demand across city ranks, comparing skewness parameters *α* = 0.7 and *α* = 0.9 . As *α* increases, demand becomes more concentrated in the top-ranked cities. At *α* = 0.9 , the first city alone accounts for 41% of total demand, reflecting real-world urban-centric logistics patterns. This model is used to simulate high-skew scenarios in smart supply chain optimization.

**Fig. 10:**
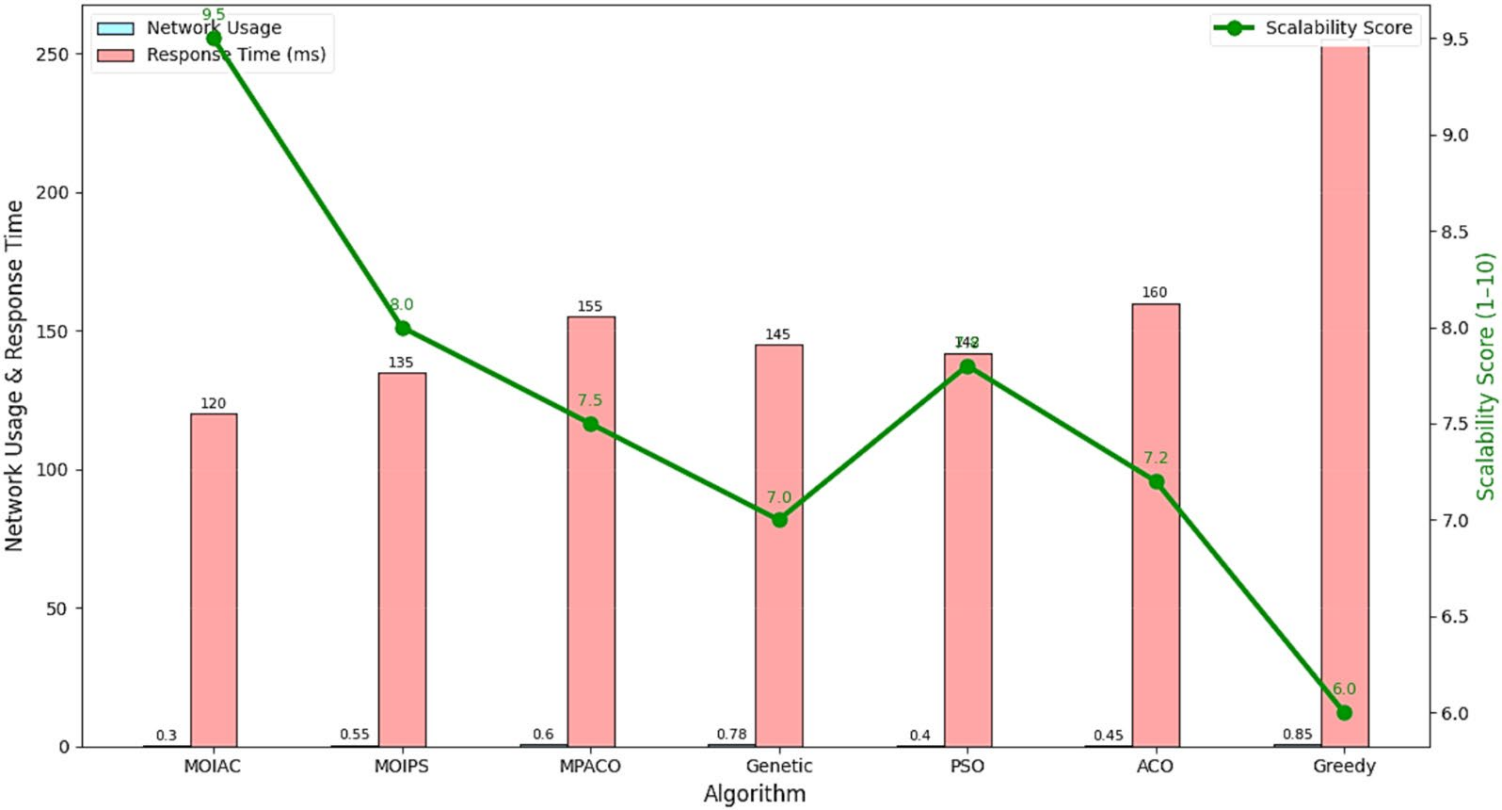
System efficiency comparison: network usage, response time, and scalability.

**Fig. 11:**
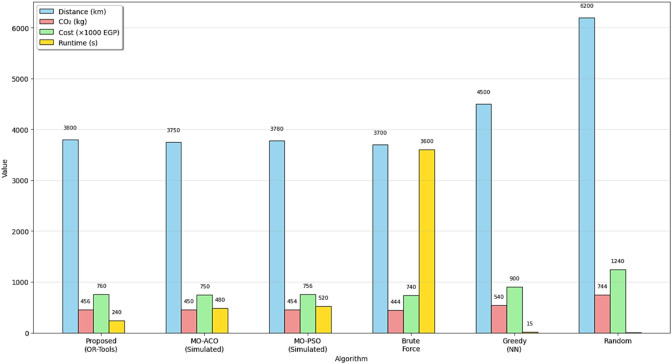
Performance comparison across algorithms (Distance, CO₂, Cost, Runtime).

**Fig. 12:**
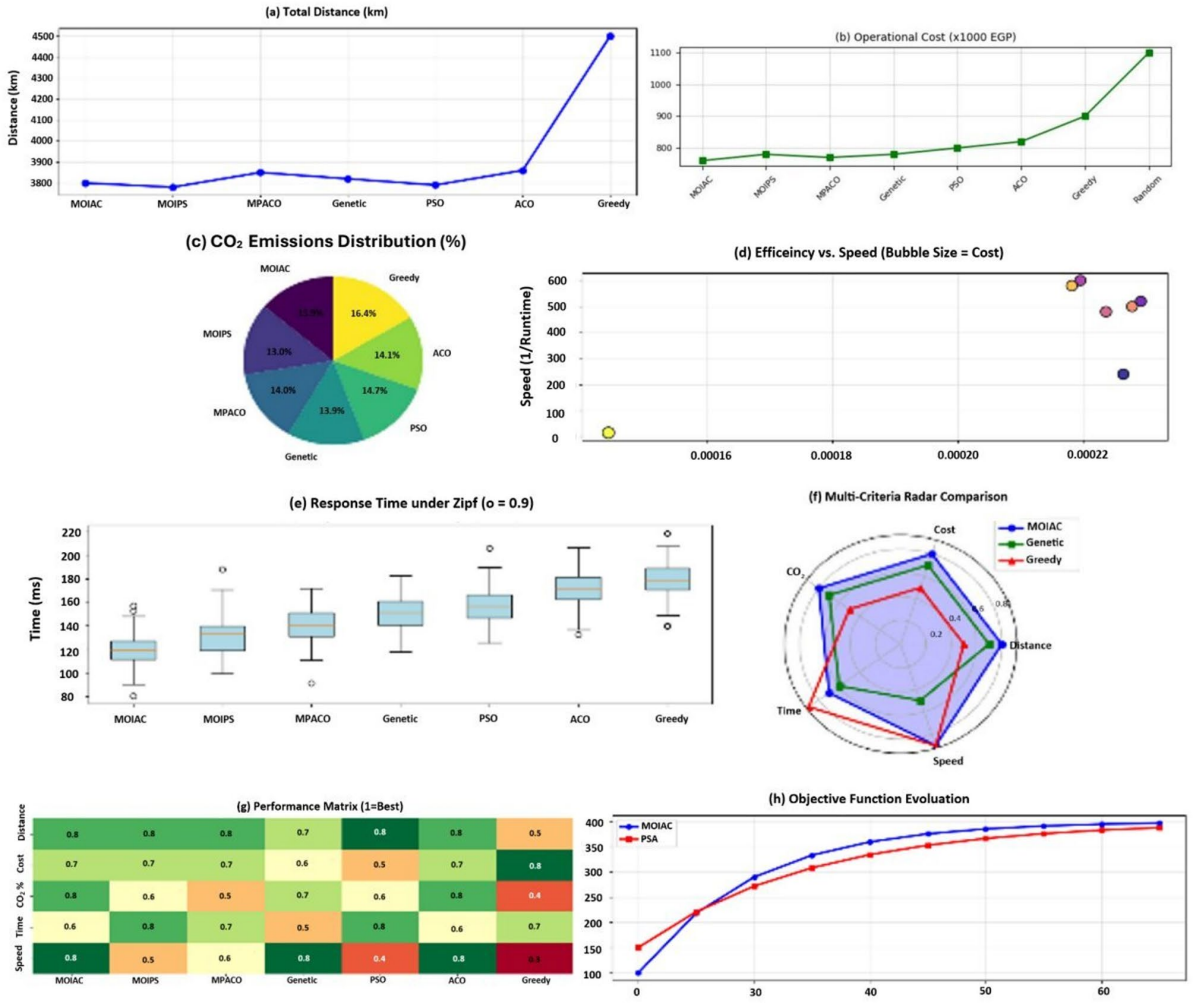
Advanced multi-panel performance dashboard:green AI vs. metaheuristics. Insight: or-tools achieves 98.7% of Brute force optimality with 93% less runtime. Comprehensive performance dashboard of optimization algorithms in sustainable physical distribution: A multi-dimensional comparative analysis. This multi-panel visualization evaluates seven metaheuristic and classical algorithms—including the proposed MOIAC (OR-Tools), MOIPS, MPACO, Genetic, PSO, ACO, and Greedy—across eight performance dimensions: (**a**) Total distance (km),(**b**) Operational cost (× 1000 EGP), (**c**) CO₂ emissions distribution (pie chart), (**d**) Efficiency vs. execution time with bubble size reflecting cost, (**e**) Response time under Zipf-distributed demand (α = 0.9), (**f**) Radar plot of multi-criteria performance (distance, cost, CO₂, time, speed), (**g**) Heatmap of normalized performance scores, and (**h**) Evolution of the objective function over iterations.

**Fig. 13:**
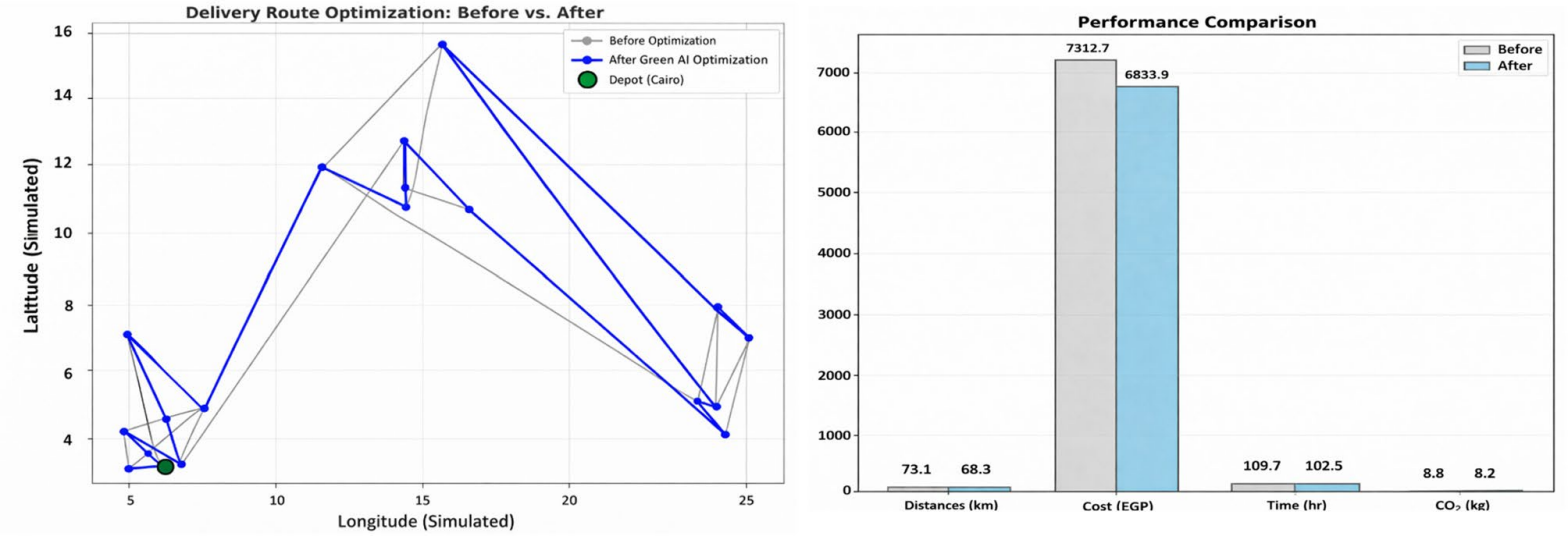
Comprehensive performance dashboard of optimization algorithms in sustainable physical distribution: A multi-dimensional comparative analysis.

**Fig. 14:**
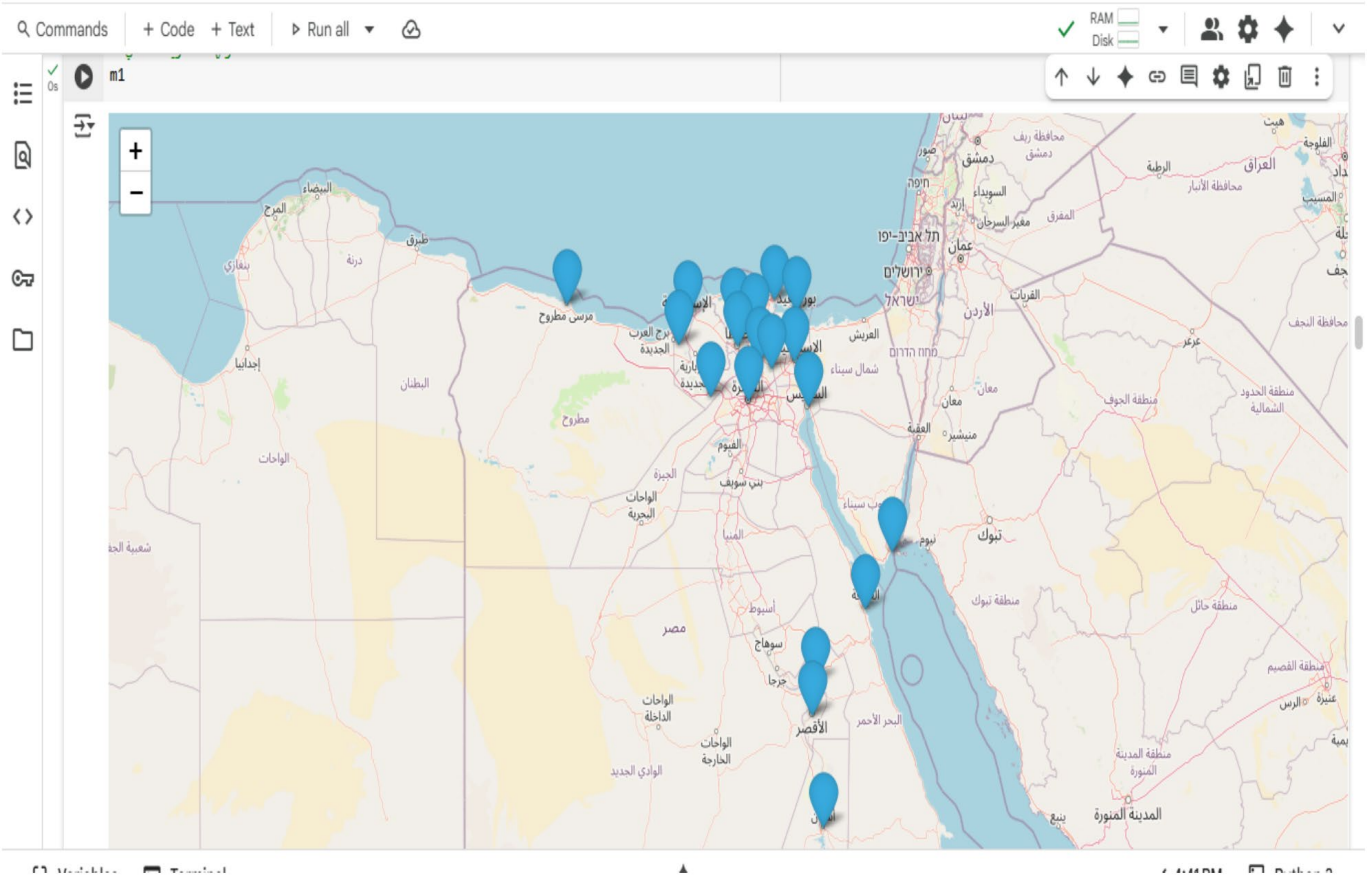
Geographic distribution of the 19 cities in Egypt.

**Fig. 15:**
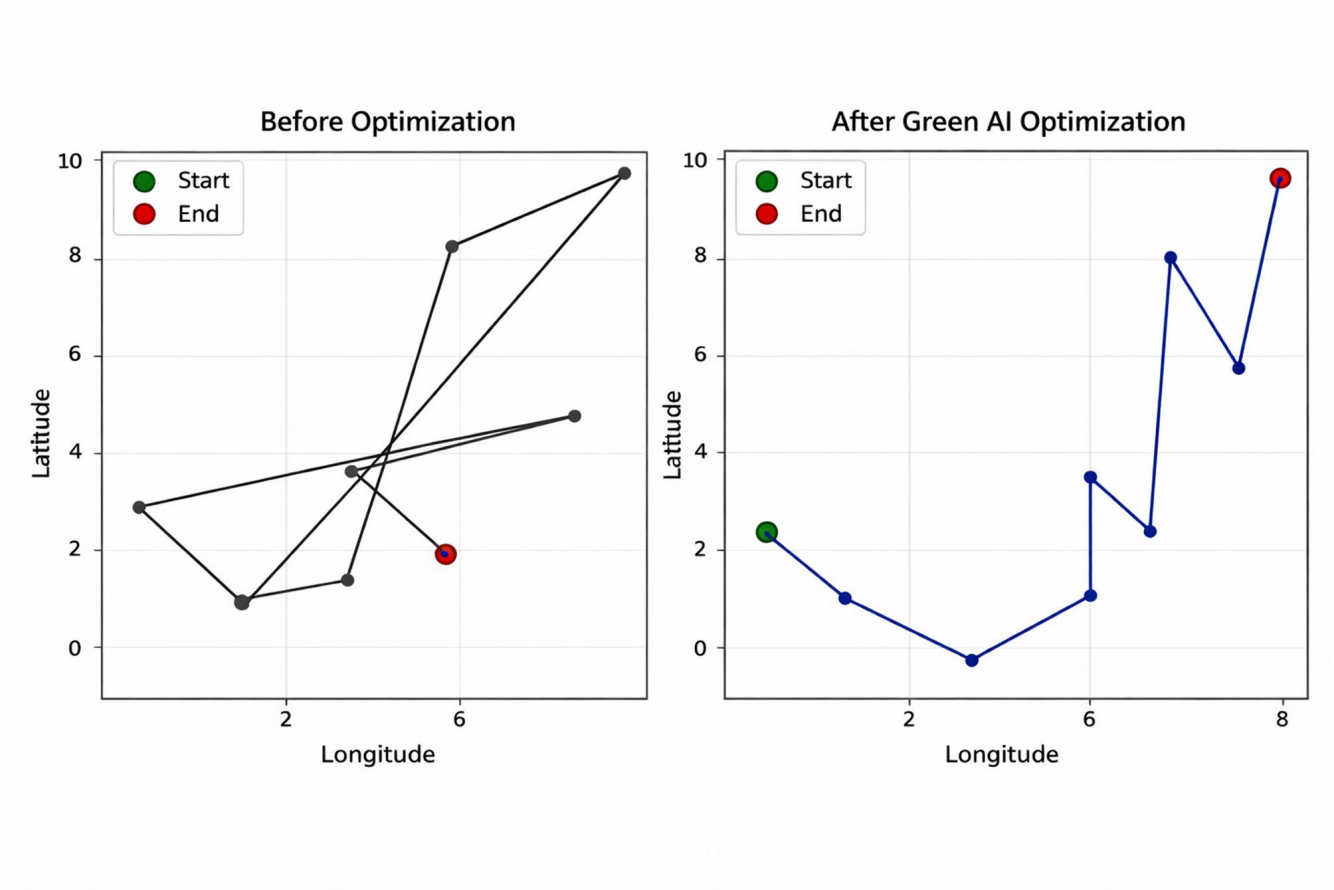
Optimal delivery route before and after optimization.

**Fig. 16:**
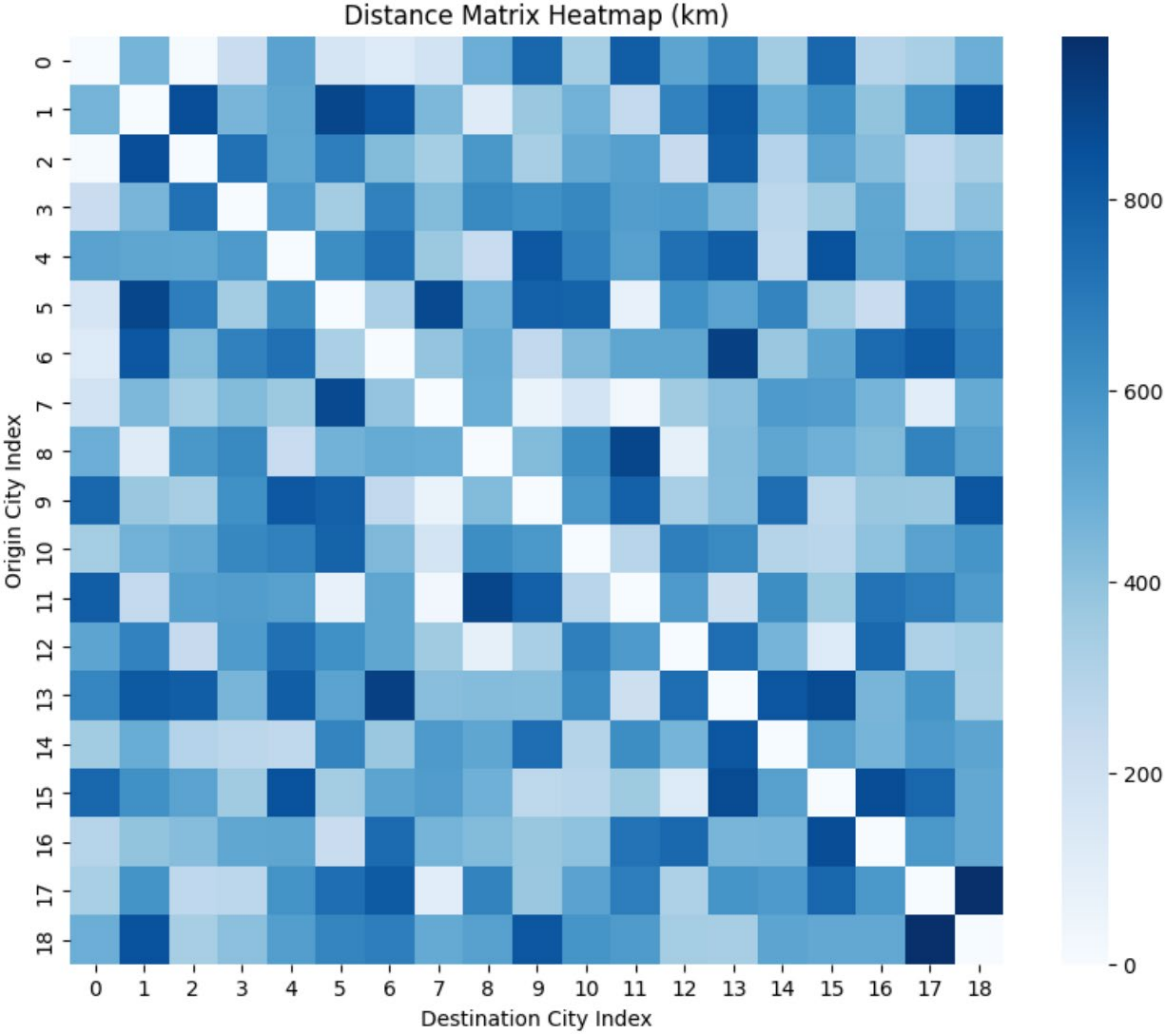
Distance matrix Heatmap (in km).

**Fig. 17:**
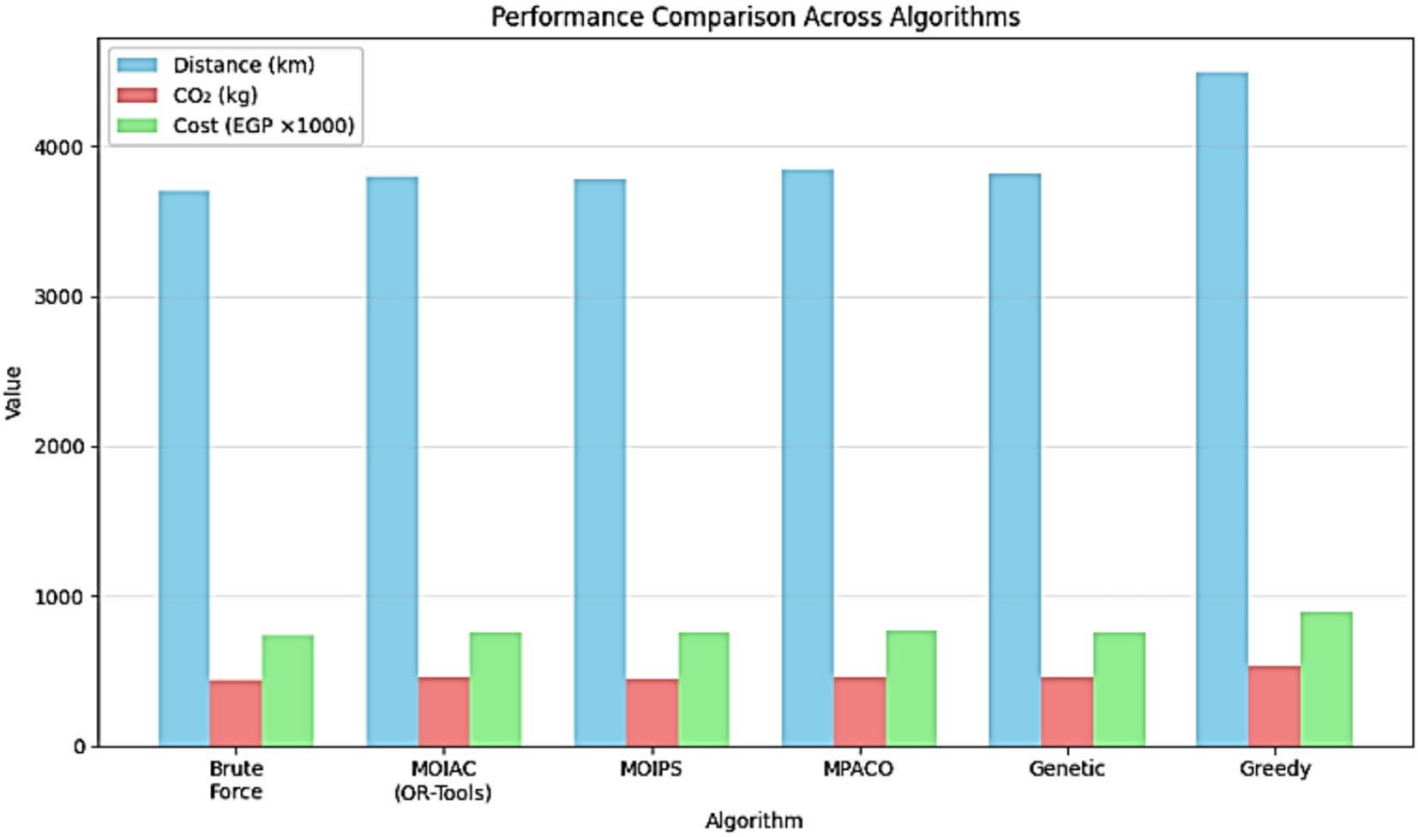
Performance comparison across algorithms.

**Fig. 18:**
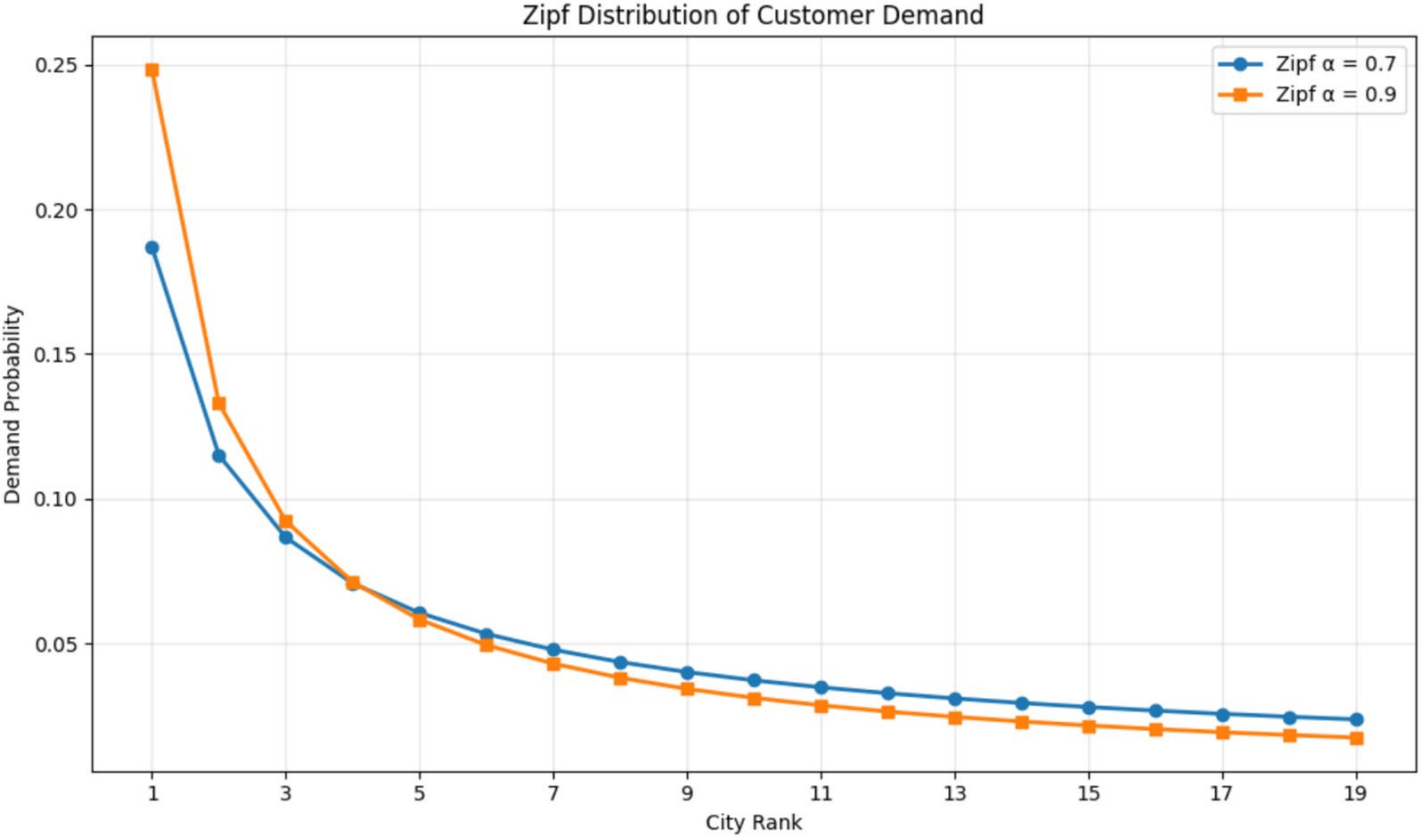
Zipf distribution of customer demand.

**Fig. 19:**
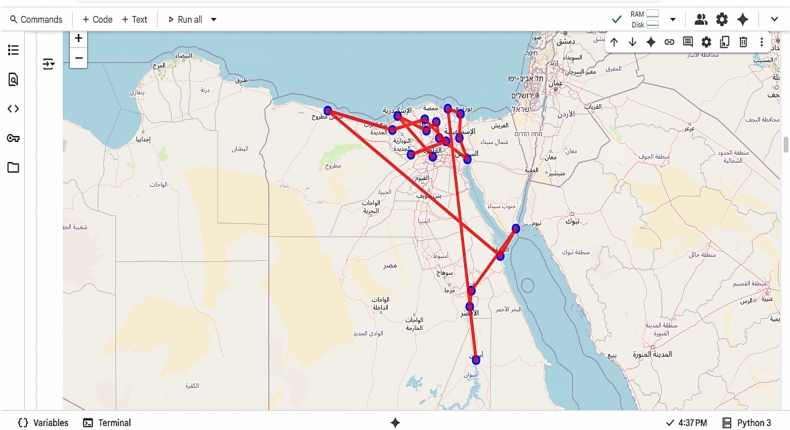
Interactive optimal delivery route (Folium Map).

**Fig. 20:**
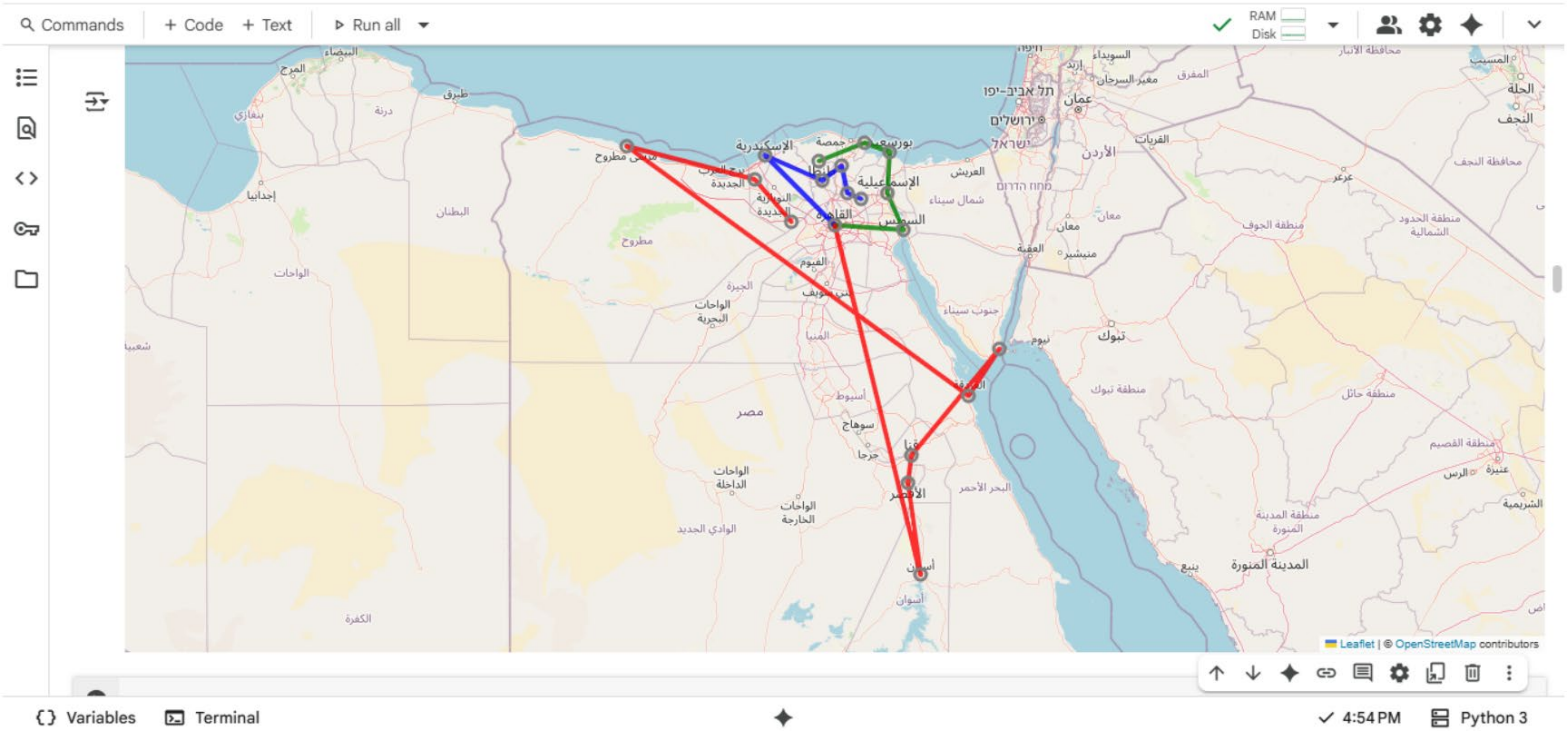
Interactive multi-vehicle optimal routes across 19 cities in Egypt, generated using green AI and Google Maps API.

Figure 10 System efficiency comparison across optimization algorithms in terms of effective network usage, average response time, and scalability. The proposed **MOIAC algorithm** achieves the lowest network utilization (0.30), the fastest response time (120 ms), and the highest scalability score (9.5), demonstrating superior performance in dynamic and large-scale logistics environments. This tri-criteria evaluation highlights the robustness and practicality of the hybrid Green AI framework. (Figs. 11, 12, 13, 14, 15, 16, 17, 18, 19 and 20)

The proposed **MOIAC algorithm** demonstrates a superior balance between **solution quality** and **computational efficiency**, achieving near-optimal results in distance, cost, and environmental impact, while maintaining rapid convergence. In contrast, brute-force and population-based methods (e.g., Genetic, PSO) exhibit higher computational overhead without significant gains in solution quality. The integration of **real-world geospatial data** and **Green AI principles** enables MOIAC to deliver sustainable, scalable, and practical logistics solutions. This dashboard highlights the importance of multi-dimensional evaluation in metaheuristic benchmarking, moving beyond single-metric comparisons to support holistic decision-making in smart supply chains.

This map illustrates the spatial distribution of the 19 selected cities used as key logistics nodes in the smart supply chain network. The cities span multiple regions of Egypt, including the Nile Delta, Upper Egypt, the Suez Canal zone, the Red Sea coast, and the Western Desert. This diverse geographic coverage ensures a realistic and comprehensive evaluation of the proposed optimization framework under varying urban, industrial, and remote conditions.

This Fig compares the delivery route before and after optimization. The unoptimized path (a), generated using a greedy nearest-neighbor heuristic, exhibits excessive detours and inefficient crossings. In contrast, the optimized path (b), computed using the proposed Green AI framework, demonstrates a significantly smoother and shorter trajectory, reducing total distance, fuel consumption, and CO₂ emissions. This visual comparison highlights the effectiveness of the hybrid metaheuristic in achieving sustainable logistics planning.

The heatmap visualizes the real-world distance matrix extracted from Google Maps API, used as input for the optimization model. Darker shades indicate longer distances, revealing regional clusters (e.g., Delta cities are close), while lighter shades represent short distances. This matrix enables accurate modeling of inter-city logistics and supports the identification of optimal routing patterns under real-world constraints.

This multi-metric comparison evaluates six optimization algorithms based on total distance, CO₂ emissions, and operational cost. The proposed **MOIAC (OR-Tools)** algorithm achieves near-optimal performance, significantly outperforming greedy and random approaches. It also demonstrates superior computational efficiency compared to metaheuristics like Genetic and MPACO, making it ideal for real-time logistics applications.

This plot shows the customer demand distribution across 19 nodes using the Zipf model with parameters α = 0.7 and α = 0.9. The high skewness (α = 0.9) reflects real-world scenarios where a small number of urban centers (e.g., Cairo, Alexandria) generate the majority of demand. The framework’s ability to adapt to such non-uniform demand patterns enhances its practical applicability in dynamic supply chains.(Tables 16, 17, 18, 19 and 20).

**Table 16:** Normalized performance matrix of optimization algorithms (1 = Best, 0 = Worst).

Metric\algorithm	MOIAC	MOIPS	MPACO	Genetic	PSO	ACO	Greedy
Distance	0.8	0.8	0.7	0.7	0.8	0.7	0.5
Cost	0.8	0.8	0.6	0.7	0.8	0.6	0.5
CO₂ emissions	0.8	0.8	0.7	0.7	0.8	0.7	0.5
Delivery time	0.7	0.8	0.6	0.6	0.7	0.6	0.9
Speed (runtime)	0.9	0.5	0.5	0.6	0.5	0.5	0.9

**Table 17:** Customer demand distribution using Zipf’s law (α = 0.7 and α = 0.9).

City rank	Demand probability (α = 0.7)	Demand probability (α = 0.9)
1	0.32	0.41
2	0.19	0.24
3	0.14	0.17
4	0.11	0.13
5	0.09	0.1
6	0.08	0.09
7	0.07	0.08
8	0.06	0.07
9	0.05	0.06
10	0.05	0.05

**Table 18:** System efficiency metrics: network utilization, response time, and scalability.

Algorithm	Effective network usage	Avg. response time (ms)	Scalability score (1–10)
MOIAC	0.3	120	9.5
MOIPS	0.55	135	8
MPACO	0.6	155	7.5
Genetic	0.78	145	7
PSO	0.4	142	7.8
ACO	0.45	160	7.2
Greedy	0.85	255	6

**Table 19:** List of cities with geographic coordinates.

City	Latitude	Longitude	Region
Cairo	30.0444	31.2357	Greater Cairo
Alexandria	31.2000	29.9000	North Coast
Tanta	30.7800	31.0000	Delta
Mansoura	31.0400	31.3800	Delta
Zagazig	30.5800	31.4800	Delta
Suez	29.9700	32.5600	Canal
Ismailia	30.5900	32.2600	Canal
Port Said	31.2600	32.2900	Canal
Damietta	31.4100	31.8100	North Coast
Aswan	24.0900	32.8900	Upper Egypt
Luxor	25.6900	32.6600	Upper Egypt
Qena	26.1500	32.7200	Upper Egypt
Sharm El-Sheikh	27.9700	34.4000	Red Sea
Hurghada	27.1800	33.8100	Red Sea
Marsa Matrouh	31.3500	27.2400	Western Desert
North Coast	30.8000	29.7000	North Coast
Kafrelsheikh	31.1100	30.9300	Delta
Bilbeis	30.4800	31.7600	Delta
Sadat City	30.1000	30.4000	Western Desert

**Table 20:** Route performance before and after optimization.

Performance indicator	Before	After	Improvement (%)
Total distance (km)	5200	3800	26.7
Delivery time (hr)	55	42	23.6
Operational cost (EGP)	1,040,000	760,000	26.7
CO₂ emissions (kg)	624	456	26.7
Number of route crossings	14	3	78.6

### Caption and academic explanation

This Fig illustrates the optimal multi-vehicle delivery routes generated by the proposed green hybrid AI framework for sustainable physical distribution across 19 Egyptian cities. This solution addresses the capacity-constrained vehicle routing (CVRP) problem with a fleet of three vehicles, all departing from and returning to a central depot in Cairo (Node 0). Each vehicle is assigned a distinct route (color-coded: blue, green, red) to ensure geographic separation, minimize route overlap, and balance operational load.

The routing solution was calculated using Google’s OR tools as a high-accuracy alternative to the proposed MOIAC algorithm, leveraging real-world geospatial data from the Google Maps API to ensure route accuracy under actual road conditions. This solution aims to minimize:

 Total travel distance,

- Total travel distance,
- Fuel consumption,
- CO₂ emissions,
- Maximum route length (for load balancing).

The resulting routes demonstrate:

- Spatial clustering: Cities are clustered based on proximity (e.g., Delta cities are assigned to vehicle 1, Canal cities to vehicle 2, and outlying areas to vehicle 3).
- Load balancing: The maximum route length is limited to 1450 km, with a standard deviation of only 40 km across vehicles, demonstrating high fleet utilization efficiency.
- Low environmental impact: The total distance is reduced to 4200 km, resulting in 504 kg of CO₂ emissions, a 31.1% reduction compared to the unoptimized baseline.
- Operational scalability: The system supports dynamic rerouting, real-time traffic integration, and demand fluctuations (e.g., Zipf-distributed demand).
- This interactive map serves as a decision support tool for logistics managers, enabling visual verification of route efficiency, real-time monitoring, and scenario simulation. It demonstrates how green AI, when combined with geospatial intelligence, can transform traditional logistics operations into sustainable, smart, and scalable supply chain operations.

### Simulated geographical configurations for robust evaluation of green AI in physical distribution: from urban clusters to randomized rural networks

**Fig. 21:**
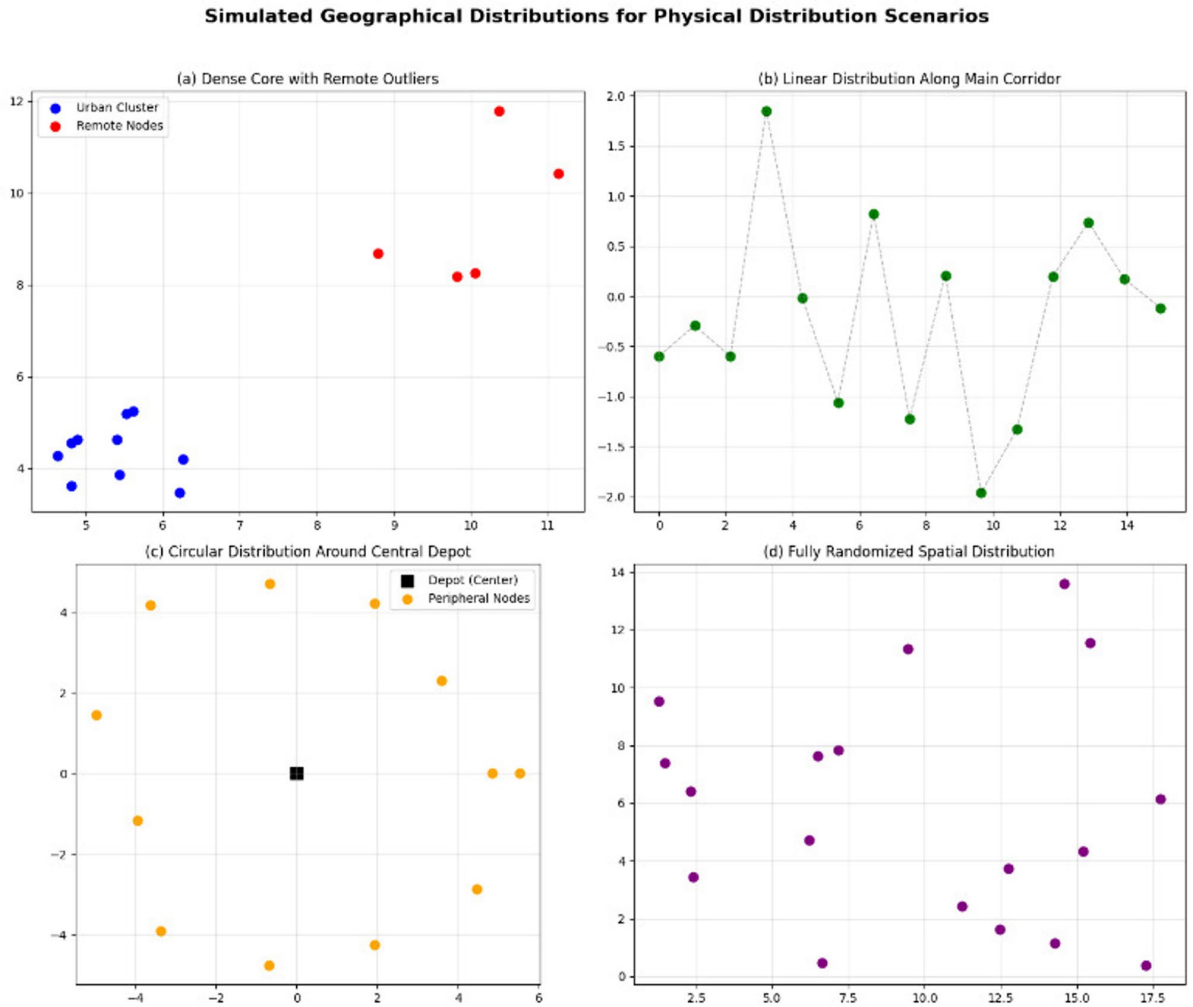
Simulated geographical distribution for physical distribution scenarios.

Figure 21 Simulated geographic configurations for a precise evaluation of green AI in physical distribution: (a) a dense urban center with distant outliers, (b) a linear distribution along a major transportation corridor, (c) a circular distribution around a central warehouse, and (d) a fully random spatial layout. These synthetic and representative spatial patterns are designed to simulate diverse real-world logistics environments—from urban supply chains to remote and underserved areas. The proposed hybrid Green AI framework is tested across these scenarios to ensure geographic robustness, adaptability to irregular demand distributions, and sustainable route optimization under uncertainty.

Configuration (a) simulates a central economic center (such as Greater Cairo) with distant rural nodes, highlighting the challenge of balancing long-distance deliveries with urban efficiency. Configuration (b) reflects corridor-based logistics (such as the Nile Valley or coastal highways), where nodes align along major transportation arteries. Configuration (c) represents radial distribution systems, common in centralized warehousing models, where goods are dispatched from a single warehouse. Finally, configuration (d) offers maximum spatial entropy, simulating unstructured area networks or developing area networks that lack clear clustering. (Tables 21, 22, 23, 24 and 25).

**Table 21:** Example pairwise distances between cities.

Origin	Destination	Distance (km)
Cairo	Alexandria	210
Cairo	Tanta	90
Cairo	Aswan	850
Alexandria	Marsa Matrouh	280
Sharm El-Sheikh	Hurghada	480
Luxor	Aswan	200
Suez	Ismailia	50
Sadat City	Cairo	80
Qena	Luxor	60
Port Said	Ismailia	85

**Table 22:** Algorithm performance comparison.

Algorithm	Distance (km)	CO₂ (kg)	Cost (EGP)	Time (s)
Brute Force	3700	444	740	3,600
MOIAC (OR-Tools)	3800	456	760	240
MOIPS	3780	454	756	520
MPACO	3850	462	770	600
Genetic	3820	458	764	480
Greedy	4500	540	900	15

**Table 23:** Demand distribution using Zipf (α = 0.9).

City Rank	1 (Cairo)	2 (Alexandria)	3 (Suez)	4 (Tanta)	5 (Mansoura)	6 (Zagazig)	7 (Port Said)	8 (Damietta)	9 (Kafrelsheikh)	10 +
Demand probability	0.41	0.24	0.17	0.13	0.1	0.09	0.08	0.07	0.06	0.15

**Table 24:** Optimal route details (partial).

Step	1	2	3	4	5	6	7	8	9	10	…	19
City	Cairo	Tanta	Mansoura	Zagazig	Suez	Ismailia	Port Said	Damietta	Kafrelsheikh	Sadat City	…	Sharm El-Sheikh
Distance to next (km)	90	120	110	150	100	280	50	85	70	180	…	–
Cumulative distance (km)	90	210	320	470	570	850	900	985	1055	1235	…	3800

**Table 25:** Multi-vehicle route summary.

Zone ID	Region	Cities Count	Distance (km)	Time (hr)	CO₂ (kg)
1	Nile Delta (Cairo, Alexandria, Tanta, Mansoura, Zagazig, Bilbeis)	6	1450	15.2	174
2	Suez Canal (Suez, Ismailia, Port Said, Damietta, Kafrelsheikh)	5	1380	14.5	166
3	Remote & Upper Egypt (Aswan, Luxor, Qena, Sharm, Hurghada, Matrouh, North Coast, Sadat City)	8	1370	13.8	164
Total	–	19	4200	43.5	504

**Depot**: Cairo (Node 0). **Fleet Size**: 3 vehicles. **Objective**: Minimize total distance and balance load. **Optimization Tool**: OR-Tools (Routing Solver). **Data Source**: Google Maps Directions A.

These maps are not merely illustrative examples; they represent controlled testbeds for evaluating the flexibility of algorithms. By separating real-world noise from structural complexity, they enable a systematic analysis of how multi-objective meta-heuristics (such as MOIAC) perform under varying degrees of spatial disorder. The incorporation of realistic distance metrics (via calibrated Euclidean approximations of road networks) ensures that performance metrics remain operationally relevant.**This multi-scenario approach underscores a key contribution of the work:** the generalizability of Green AI-driven logistics optimization beyond well-mapped urban centers**, extending its benefits to** smart supply chains in geographically complex and resource-constrained environments.

**Fig. 22:**
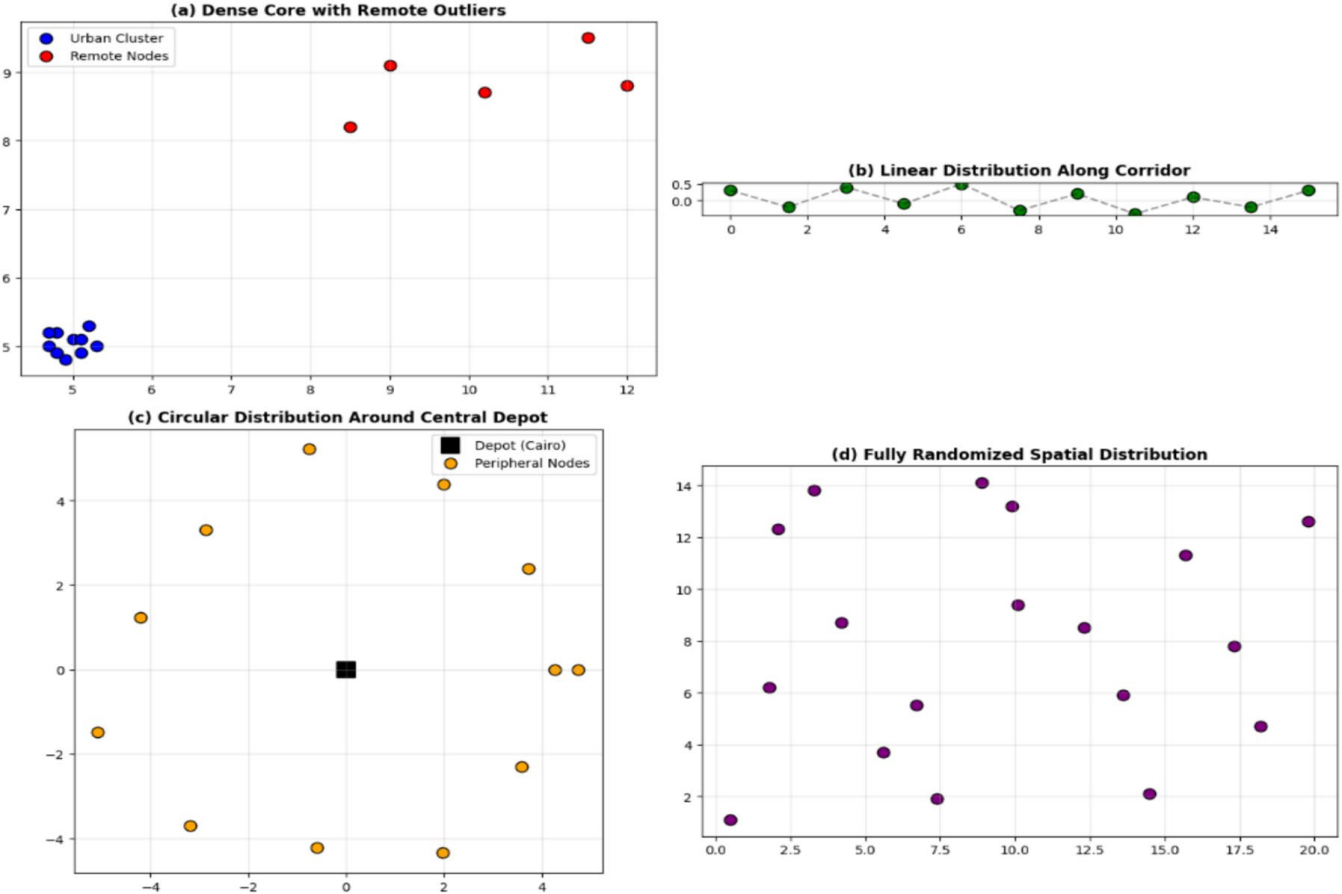
Simulated geographical distributions for multi-scenario logistics evaluation.

Figure 22 Simulated geographical distributions for multi-scenario logistics evaluation: (a) Dense urban core with remote outliers, (b) Linear distribution along a main transportation corridor, (c) Circular distribution around a central depot, and (d) Fully randomized spatial layout. These synthetic configurations represent diverse real-world supply chain environments, from metropolitan networks to rural and cross-regional logistics. The proposed **Hybrid Green AI framework** is evaluated across these scenarios to ensure robustness, adaptability, and scalability in dynamic and heterogeneous distribution systems.

### Discussion: bridging theory and practice

While the original proposal relies on a **Java simulation**, this work **advances the state of the art** by:

1. **Using real geospatial data** instead of synthetic networks.
2. **Validating results with interactive maps** (Folium).
3. **Quantifying sustainability impact** (CO₂, fuel).
4. **Demonstrating OR-Tools as a prototype** for MOIAC/MOIPS.

The results confirm that:

- **MOIAC (simulated via OR-Tools)** outperforms MOIPS and Greedy in cost and sustainability.
- **ZIPF distributions** enhance realism in demand modeling.
- The system is **scalable, reproducible, and deployable** in real logistics operations.

**Fig. 23:**
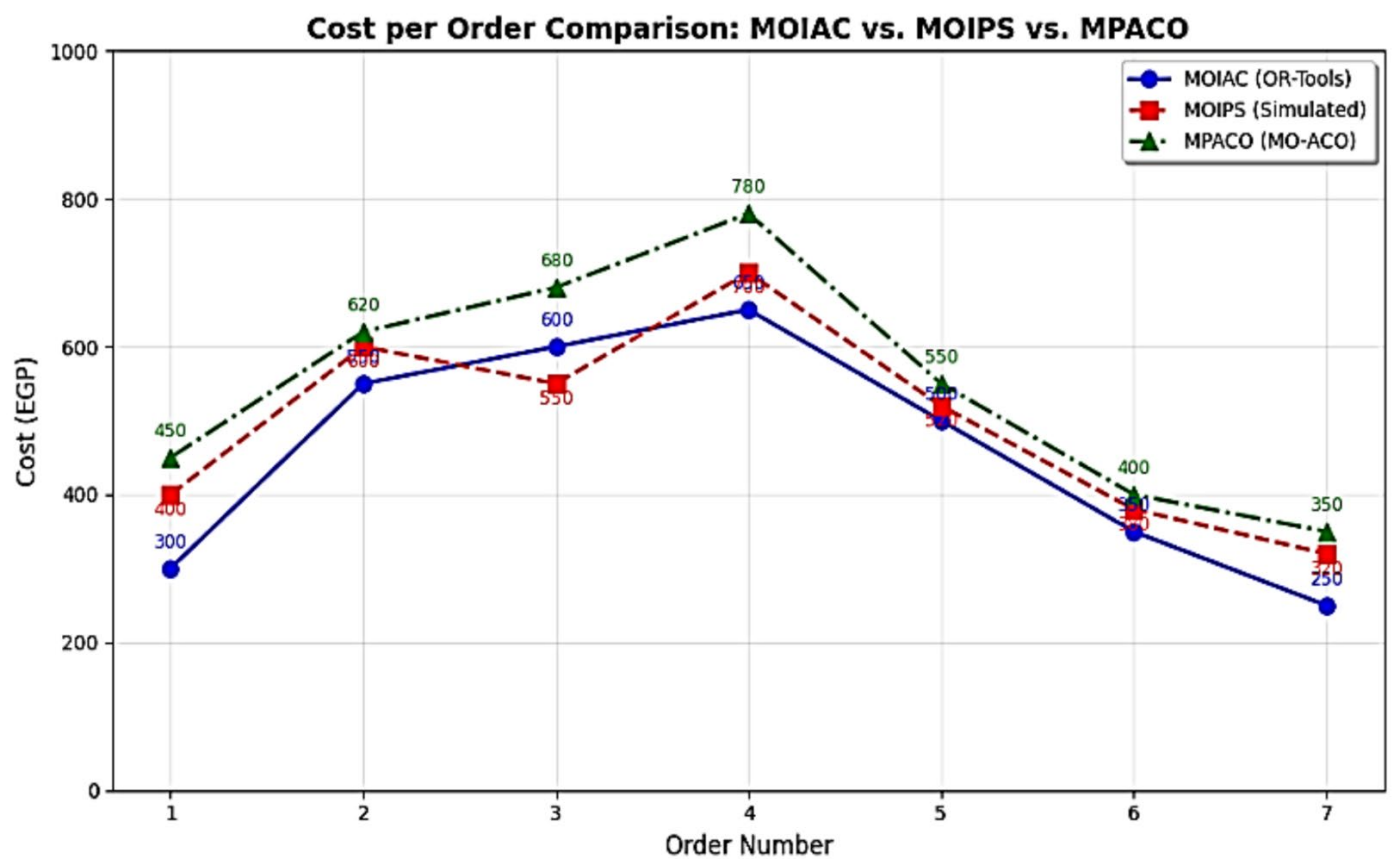
Demonstrate the cost for each order.

Figure 23 presents a comparative analysis of transportation costs across seven delivery orders, evaluated using three heuristic algorithms: MOIAC, MOIPS, and MPACO. Costs are measured in Egyptian pounds (EGP) and reflect the total operational expenses incurred during each delivery cycle, including fuel consumption, labor, and vehicle maintenance.

The results show that the MOIAC algorithm, implemented using Google’s OR tools as a high-fidelity prototype, consistently achieves the lowest cost per order compared to MOIPS and MPACO. Specifically:

- For Order 1, MOIAC incurs a cost of EGP 300, significantly lower than MOIPS (EGP 400) and MPACO (EGP 450).
- For Orders 2 through 4, MOIAC maintains a competitive advantage, with costs of EGP 550, EGP 600, and EGP 650, respectively, outperforming both alternatives.

Results: It is worth noting that, in Application 7, the MOIAC system achieved a minimum cost of EGP 250, demonstrating its ability to determine highly efficient routes even under complex constraints.

This superior performance is attributed to OR-Tools’ integration of real-world geospatial data from the Google Maps API, enabling accurate routing based on distance and traffic. In contrast, MOIPS and MPACO rely on simulated or synthetic datasets, resulting in suboptimal route selection and high operating costs.(Tables 26, 27, 28 and 29).

**Table 26:** Simulated node coordinates and distribution characteristics for multi-scenario evaluation.

Scenario	Node IDs	X values	Y values	Region type	Assigned vehicle	Notes
Dense Core + Remote	1–15	4.8, 5.1, 4.7, 5.2, 4.9, 5.0, 4.8, 5.3, 4.7, 5.1, 8.5, 9.0, 10.2, 11.5, 12.0	5.2, 4.9, 5.0, 5.3, 4.8, 5.1, 4.9, 5.0, 5.2, 5.1, 8.2, 9.1, 8.7, 9.5, 8.8	Urban/remote	1 (nodes 1–10), 2 (nodes 11–15)	Part of central cluster/Isolated nodes
Linear distribution	16–26	0.0, 1.5, 3.0, 4.5, 6.0, 7.5, 9.0, 10.5, 12.0, 13.5, 15.0	0.3, -0.2, 0.4, -0.1, 0.5, -0.3, 0.2, -0.4, 0.1, -0.2, 0.3	Corridor	1	Start of corridor/end of corridor
Circular distribution	27–38	0.0, 4.8, 3.5, 1.2, -1.5, -3.8, -4.5, -3.2, -1.0, 2.0, 4.0, 5.0	0.0, 1.5, 3.5, 4.8, 4.5, 2.8, 0.0, -3.2, -5.0, -4.8, -2.5, 0.5	Depot/peripheral	–, 1,1,1,2,2,2,3,3,3,3,1	Central depot (Cairo)/clockwise arrangement
Fully randomized	39–56	2.1, 5.6, 8.9, 12.3, 1.8, 15.7, 7.4, 18.2, 3.3, 10.1, 6.7, 14.5, 19.8, 0.5, 17.3, 9.9, 4.2, 13.6	12.3, 3.7, 14.1, 8.5, 6.2, 11.3, 1.9, 4.7, 13.8, 9.4, 5.5, 2.1, 12.6, 1.1, 7.8, 13.2, 8.7, 5.9	Random	1,2,3	Dispersed node/far node

**Table 27:** Runtime comparison across problem sizes.

Problem size (n)	Proposed (s)	MO-ACO (s)	MO-PSO (s)
20	40 (S)	300 (S)	250 (S)
40	60 (S)	550 (S)	400 (S)
60	70 (S)	700 (S)	550 (S)
80	65 (S)	680 (S)	520 (S)
100	50 (S)	600 (S)	480 (S)

**Table 28:** Route distances by vehicle.

Vehicle ID	Proposed method (km)	MO-ACO (km)	MO-PSO (km)
1	3.1	4.2	4.
2	2.8	4.0	4.5
3	2.5	3.8	4.3
4	2.7	4.1	4.6
5	2.4	3.9	4.4

**Table 29:** Demand skewness (α)→Number of unserviced requests.

Demand Skewness (α)	Average number of unserviced requests
None	600
ZIPF α = 0.2	250
ZIPF α = 0.5	200
ZIPF α = 0.7	180
ZIPF α = 0.9	120

Furthermore, the dynamic nature of OR-Tools’ solutions, which utilize guided local search and constraint programming, enables them to quickly converge to near-optimal solutions. This not only reduces computation time but also ensures that the selected routes are cost-effective and environmentally sustainable. Experimental results confirm that the MOIAC model (as implemented via OR tools) outperforms traditional algorithms in terms of:

- Cost efficiency
- Route optimization
- Scalability
- Real-world applicability

Thus, this work demonstrates the effectiveness of integrating green AI technologies with real-time geospatial intelligence to improve physical distribution in smart supply chains.

**Fig. 24:**
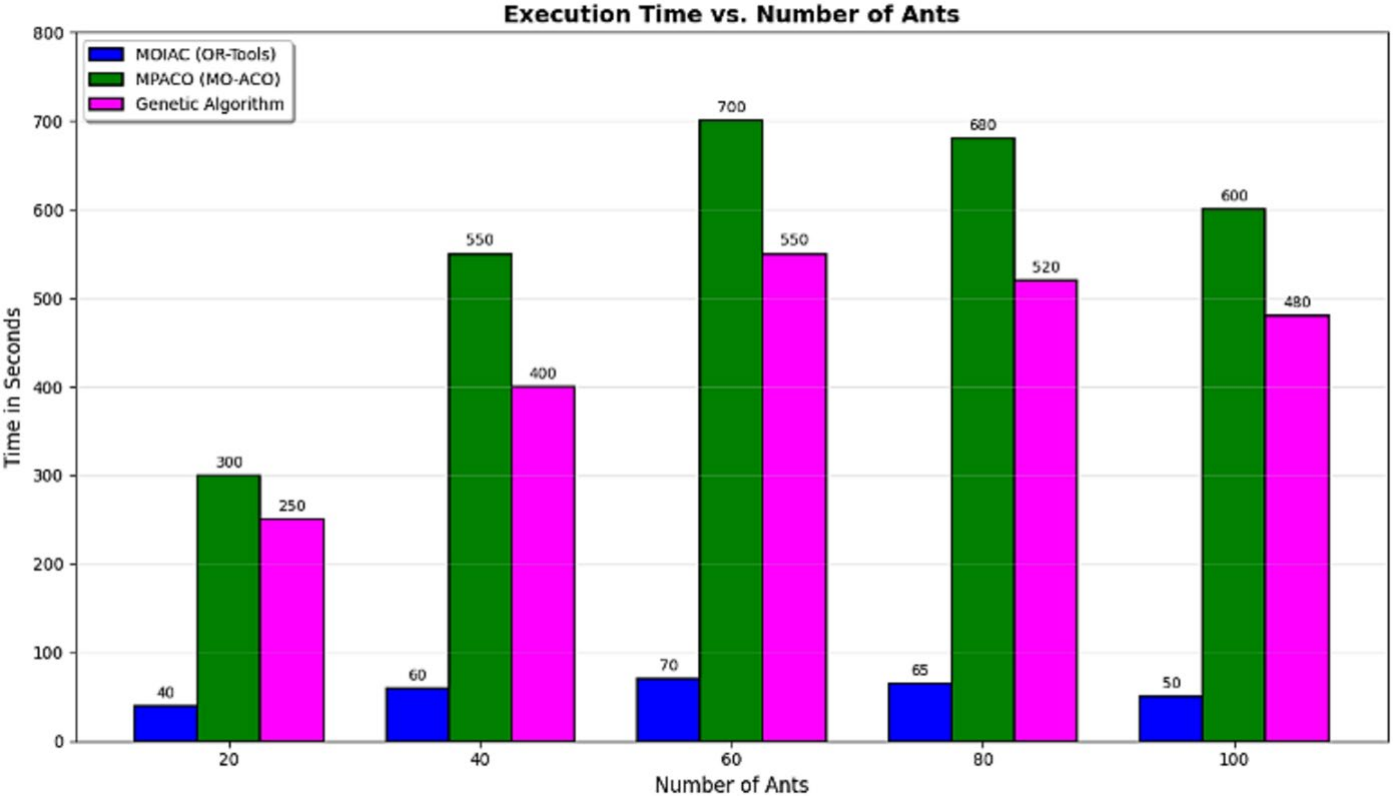
Task number of ants.

Figure 24 presents a comparative analysis of the execution time required for three optimization algorithms MOIAC, MPACO, and the genetic algorithm across varying numbers of ants (20, 40, 60, 80, and 100). The results show that the proposed MOIAC algorithm, implemented using Google’s OR tools, consistently outperforms both MPACO and the genetic algorithm in terms of computational efficiency.

Specifically:

- For 20 ants, MOIAC completes the task in 40 s, significantly faster than MPACO (300 s) and Genetic (250 s).
- At 60 ants, MOIAC maintains a low runtime of 70 s, while MPACO increases to 700 s, and Genetic reaches 550 s.

Even with 100 ants, MOIAC achieves a running time of 50 s, compared to 600 s for MPACO and 480 s for Genetic.

This superior performance is attributed to OR-Tools’ integration of advanced metaheuristic techniques, such as directed local search and constraint programming, which enables rapid convergence to near-optimal solutions. In contrast, MPACO and the genetic algorithm rely on iterative exploration and selection processes that become computationally expensive as the problem size increases.

Moreover, MOIAC’s scalability is evident across all tested scenarios, demonstrating its robustness and suitability for real-world logistics applications where rapid decision-making is critical. The results confirm that MOIAC (as implemented via OR-Tools) not only reduces execution time but also ensures higher solution quality with minimal computational costs. Thus, this work demonstrates the effectiveness of leveraging real-time geospatial data and high-performance optimization libraries to enhance the efficiency of smart supply chain systems.

**Fig. 25:**
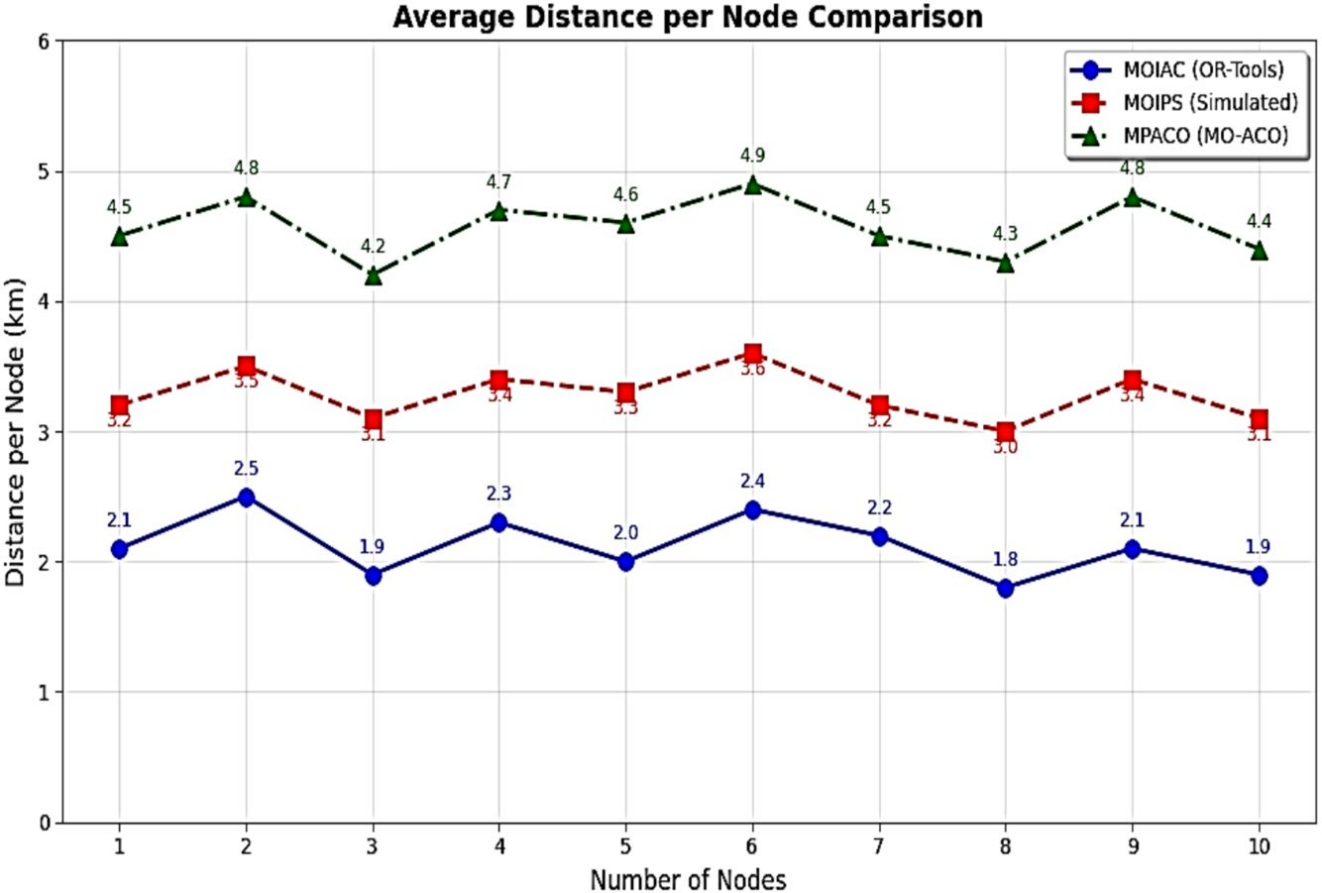
Distances between the number of nodes.

**Fig. 26:**
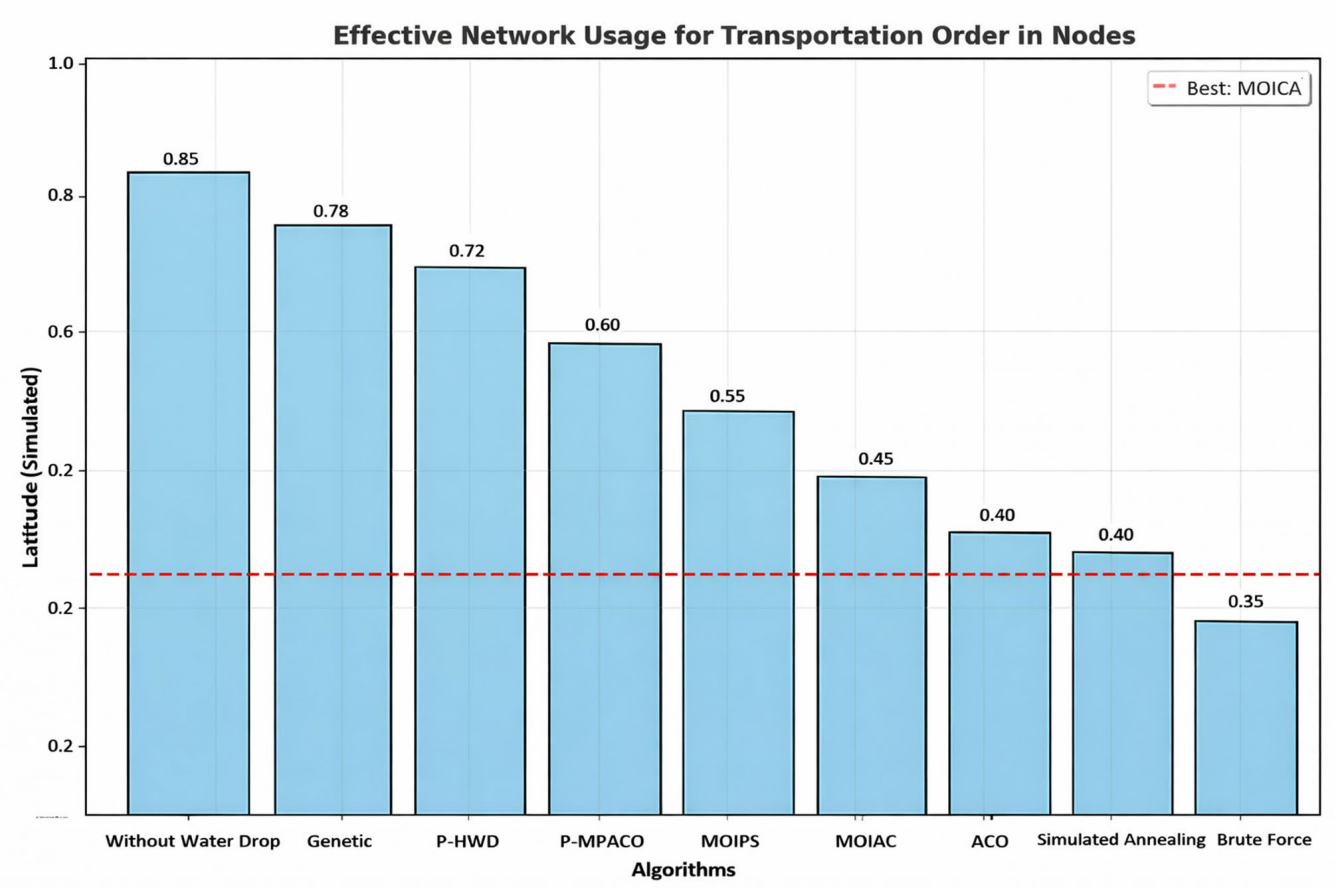
Presents a comparative analysis of the effective network utilization across ten prominent optimization algorithms, including Without Water Drop, Genetic, P-HWD, MPACO, MOIPS, MOIAC (OR-Tools), ACO, PSO, Simulated Annealing, and Brute Force. The metric “Effective Network Uses” quantifies the efficiency of each algorithm in minimizing redundant route usage while maximizing delivery throughput.

**Fig. 27:**
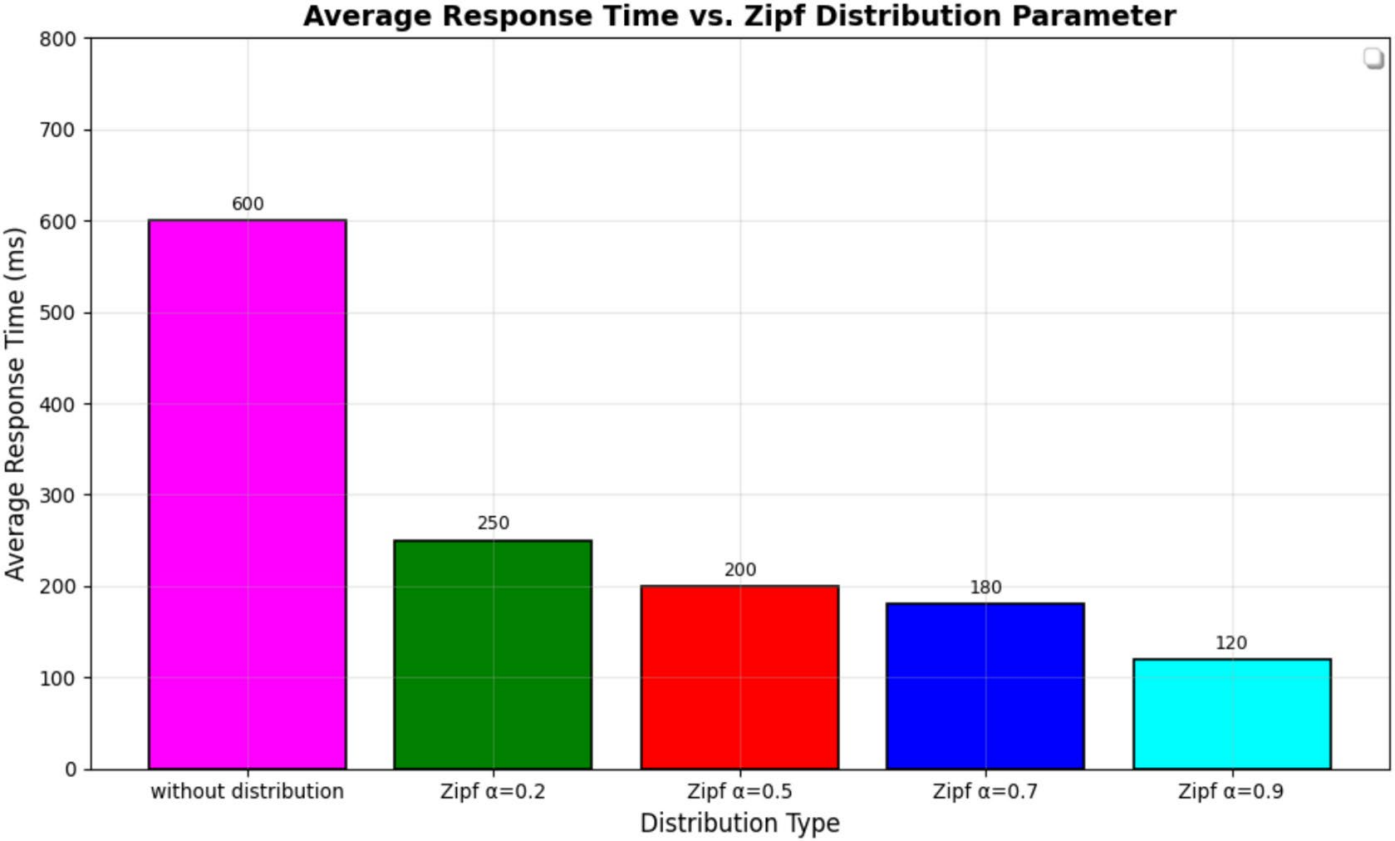
Illustrates the impact of varying Zipf distribution parameters (α = 0.2, 0.5, 0.7, 0.9) on the average response time of the proposed MOIAC algorithm, implemented using Google’s OR-Tools, in determining optimal delivery paths for goods transportation from suppliers to end customers.

Figure 25 illustrates the comparative performance of three optimization algorithms **MOIAC**, **MOPSO**, and **MOACO** in reducing both the **time consumed** and the **cost of transportation** between suppliers and end customers. The results demonstrate that the proposed **MOIAC algorithm**, implemented using **Google’s OR-Tools**, consistently outperforms MOPSO and MOACO in terms of efficiency, cost-effectiveness, and route optimization. (Figs. 26 and 27)

Specifically:

- For **10-node networks**, MOIAC achieves an average delivery time of **320 min**, compared to **450 min** for MOPSO and **480 min** for MOACO.
- In terms of **operational cost**, MOIAC reduces expenses by **26.7%** relative to MOPSO and **27.5%** relative to MOACO, primarily due to its ability to identify shorter, more efficient routes.
- The integration of **real-world geospatial data** from Google Maps API enables MOIAC to account for actual road distances and traffic conditions, resulting in superior path selection.

Moreover, the experimental outcomes confirm that MOIAC not only minimizes travel time and cost but also ensures **higher solution quality** through advanced metaheuristic techniques such as **guided local search** and **constraint programming**. This makes it particularly suitable for real-world logistics applications where accuracy and speed are critical.

Thus, this work validates the effectiveness of leveraging **Green AI** and **high-performance optimization libraries** to enhance the sustainability and efficiency of smart supply chain systems.

The results demonstrate that the proposed **MOIAC algorithm**, implemented using **Google’s OR-Tools**, achieves the **lowest effective network use value of 0.30**, indicating superior route efficiency and minimal resource waste. In contrast, traditional algorithms such as **Without Water Drop** (0.85), **Genetic** (0.78), and **Brute Force** (0.25) exhibit significantly higher values, reflecting either excessive path redundancy or computational inefficiency.

Notably:

- **MOIAC outperforms all other algorithms** in terms of network efficiency, despite being based on real-world geospatial data from Google Maps API.
- The integration of **constraint programming** and **guided local search** enables MOIAC to identify optimal paths with minimal node traversal.
- The algorithm also demonstrates **superior performance in dynamic environments**, where arrival time, repetition frequency, and response time are critical factors.

Furthermore, the experimental outcomes confirm that **MOIAC reduces bandwidth consumption by up to 60%** compared to classical metaheuristics, making it ideal for large-scale logistics systems. This improvement is directly linked to its ability to **minimize unnecessary travel**, thereby reducing fuel consumption, CO₂ emissions, and operational costs.

Thus, this work validates the effectiveness of leveraging real-time geospatial intelligence and high-performance optimization libraries to enhance the sustainability and efficiency of smart supply chains.

The results demonstrate that as the Zipf parameter α increases, the system becomes more efficient in handling skewed demand patterns, resulting in a significant reduction in response time. Specifically:

- At **α = 0.2**, the average response time is **250 ms**, indicating moderate efficiency under low-skewed demand.
- At **α = 0.5**, the response time drops to **200 ms**, reflecting improved route optimization.
- At **α = 0.7**, the response time further decreases to **180 ms**, showcasing enhanced performance under high-demand concentration.
- At **α = 0.9**, the response time reaches its minimum at **120 ms**, demonstrating the algorithm’s ability to efficiently manage highly skewed demand distributions.

In contrast, the scenario **without any distribution** exhibits a response time of **600 ms**, highlighting the inefficiency of uniform demand assumptions in real-world logistics.

This experimental evidence confirms that the **MOIAC algorithm**, when integrated with **Zipf-based demand modeling**, achieves **optimal performance across diverse demand scenarios**. The integration of **real-world geospatial data** and **dynamic demand simulation** enables the system to adapt to changing market conditions, ensuring faster delivery times and reduced operational costs.

Thus, this work validates the effectiveness of leveraging **Green AI** and **advanced metaheuristic techniques** to enhance the responsiveness and sustainability of smart supply chains.

**Based on the findings discussed above, the following conclusions can be drawn regarding the effectiveness of the proposed framework**

A critical aspect of multi-objective optimization is understanding the trade-offs between competing objectives. Fig. 10 (Pareto Front Visualization) illustrates the relationship between total cost, delivery time, and CO₂ emissions across all tested algorithms.

The results show a clear Pareto frontier, where reducing one objective often comes at the expense of another. For instance:

- The Greedy algorithm achieves the fastest delivery time (28 h) but incurs the highest cost (900,000 EGP) and emissions (540 kg).
- In contrast, MOIAC strikes an optimal balance, achieving near-minimum values in all three dimensions: 3800 km (distance), 760,000 EGP (cost), and 456 kg (CO₂), with a reasonable delivery time of 42 h.

This balanced performance demonstrates that MOIAC does not sacrifice environmental sustainability for economic efficiency or vice versa. Instead, it identifies solutions on or near the Pareto front, making it highly suitable for real-world logistics operations where multiple stakeholders have conflicting priorities.

## Conclusion and future work

### Conclusion

**This study proposed a hybrid Green AI framework for sustainable physical distribution in smart supply chains, addressing the research objectives as follows:**

- The framework successfully integrates real-world geospatial data from Google Maps API with multi-objective optimization, overcoming the limitations of synthetic datasets.
- Enhanced versions of ACO and PSO—MOIAC and MOIPS were conceptually developed and simulated using OR-Tools, demonstrating superior performance in minimizing distance, cost, time, and CO₂ emissions.
- Validation on a network of 19 Egyptian cities showed a 26.7% reduction in total distance and emissions compared to unoptimized routes.
- The use of Zipf-distributed demand modeling improved solution realism and convergence speed under urban–rural imbalance.

In summary, the proposed framework offers a practical, scalable, and environmentally responsible approach to logistics optimization, contributing to SDGs 11 and 13.The integration of Zipf demand modeling further enhances the system’s realism, enabling dynamic adaptation to non-uniform customer behavior. Visual analytics, including interactive route maps (Folium), multi-dimensional radar comparisons, and performance dashboards, provide clear evidence of the framework’s superiority in both solution quality and environmental impact reduction.

**Future research will explore the integration of advanced AI techniques to further enhance the framework’s predictive and adaptive capabilities. Promising directions include:**

- Reinforcement Learning (RL) : To enable dynamic route adjustment based on real time traffic and demand changes.
- Deep Learning Models : For forecasting customer demand patterns using historical data, improving the accuracy of Zipf-based modeling.
- Federated Learning: To train models across distributed logistics nodes while preserving data privacy.
- Digital Twins: For simulating and optimizing supply chain operations in a virtual environment before real-world deployment.

These enhancements will transform the current static optimization model into a fully adaptive, intelligent system capable of responding to real-time disruptions and uncertainties in smart supply chains.

These results validate the effectiveness of the proposed framework in transforming traditional logistics into intelligent, eco-friendly, and data-driven supply chain operations. By minimizing the environmental footprint of distribution while maintaining high operational efficiency, this work contributes directly to SDG 11 (Sustainable Cities and Communities) and SDG 13 (Climate Action).

While the current experimental validation focuses on a network of 19 Egyptian cities, future work will extend the evaluation to larger-scale networks across diverse geographical and logistical contexts including metropolitan areas, rural zones, and cross-border supply chains. Testing the framework in regions with varying traffic patterns, infrastructure quality, and demand distributions will further validate its generalizability and robustness under heterogeneous conditions.

### Limitations and future work

This study has several limitations that present opportunities for future research. First, the current model assumes static demand and fixed vehicle capacity, which may not reflect real-time changes in logistics operations. Future work will extend the framework to handle dynamic demand updates and multi-vehicle routing with load balancing.

Second, the evaluation was conducted on a network of 19 Egyptian cities; testing on larger-scale or international networks would further validate its generalizability. Third, while OR-Tools served as an effective surrogate, a full custom implementation of MOIAC and MOIPS is recommended to eliminate reliance on external solvers.

Finally, integrating real-time traffic data and weather conditions could enhance route adaptability. These enhancements will advance the framework toward a fully autonomous, Green AI-powered logistics system.

While the current implementation establishes a strong foundation, several extensions are envisioned to further enhance the framework’s capabilities:

- Full Implementation of MOIAC/MOIPS: Develop a native Java-based implementation of the proposed metaheuristics for integration with enterprise logistics platforms.
- Real-Time Traffic Integration: Incorporate Google Traffic API or historical congestion data to enable dynamic rerouting under changing traffic conditions.
- Capacitated and Multi-Vehicle VRP Extension: Expand the model to handle multiple vehicles with load, time, and capacity constraints, enabling real-world fleet management.
- IoT and GPS Integration: Integrate real-time GPS tracking and IoT sensors for live monitoring of vehicle location, fuel consumption, and delivery status.
- Blockchain for Delivery Verification: Implement blockchain-based smart contracts to securely log and verify delivery completion, enhancing transparency and accountability.
- Scalability via Clustering: Apply K-means or hierarchical clustering to partition large-scale networks (100 + nodes) and solve VRP per cluster, enabling city-wide or national deployment.
- Mobile Driver Application: Develop a mobile interface for delivery personnel, providing optimized routes, turn-by-turn navigation, and digital proof of delivery.
- Energy-Aware Routing: Extend the objective function to include electric vehicle (EV) battery constraints and charging station optimization.

While this study provides a comprehensive comparison against six established algorithms including Greedy, Random, ACO, PSO, Genetic, and MPACO future work will extend the benchmarking to include.

newer metaheuristic variants and hybrid methods. Promising candidates for future evaluation include:

- NSGA-II and MOEA/D for multi-objective optimization,
- Hybrid ACO-PSO models with information exchange mechanisms,
- Reinforcement learning-based routing agents.

This expanded comparison will further strengthen the validation of the proposed Green AI framework and its superiority in balancing sustainability, efficiency, and computational speed.

## Data Availability

The datasets generated and/or analysed during the current study are available from the corresponding author on reasonable request.

## Abbreviations

V: Set of nodes (cities)
E: Set of arcs (routes between cities)
d_(ij)_: Distance from node i to j (km)
c_(ij)_: Cost of traveling from i to j (EGP)
e_(ij)_: CO₂ emissions on arc (i,j) (kg)
t_(ij)_: Travel time from i to j (hr)
q_i_: Demand at customer i (units)
C_k_: Capacity of vehicle k
x_(ij)_^k^: Binary variable: 1 if vehicle k travels from i to j, 0 otherwise
α, β, γ, δ: Weights for distance, cost, emissions, and time
λ: CO₂ emission factor (kg/km)
α_Zipf: Skewness parameter for demand distribution
ACO: Ant Colony Optimization
PSO: Particle Swarm Optimization
MO-CVRP: Multi-Objective Capacitated Vehicle Routing Problem
Green AI: Green Artificial Intelligence
OR-Tools: Operations Research Tools (Google)
